# Microbial Degradation of Naphthalene and Substituted Naphthalenes: Metabolic Diversity and Genomic Insight for Bioremediation

**DOI:** 10.3389/fbioe.2021.602445

**Published:** 2021-03-09

**Authors:** Balaram Mohapatra, Prashant S. Phale

**Affiliations:** Department of Biosciences and Bioengineering, Indian Institute of Technology Bombay, Mumbai, India

**Keywords:** biodegradation, metabolic pathways, genetic diversity, gene transfer mechanisms, cellular responses and evolution, bioremediation, naphthalene, substituted naphthalenes

## Abstract

Low molecular weight polycyclic aromatic hydrocarbons (PAHs) like naphthalene and substituted naphthalenes (methylnaphthalene, naphthoic acids, 1-naphthyl *N*-methylcarbamate, etc.) are used in various industries and exhibit genotoxic, mutagenic, and/or carcinogenic effects on living organisms. These synthetic organic compounds (SOCs) or xenobiotics are considered as priority pollutants that pose a critical environmental and public health concern worldwide. The extent of anthropogenic activities like emissions from coal gasification, petroleum refining, motor vehicle exhaust, and agricultural applications determine the concentration, fate, and transport of these ubiquitous and recalcitrant compounds. Besides physicochemical methods for cleanup/removal, a green and eco-friendly technology like bioremediation, using microbes with the ability to degrade SOCs completely or convert to non-toxic by-products, has been a safe, cost-effective, and promising alternative. Various bacterial species from soil flora belonging to *Proteobacteria* (*Pseudomonas*, *Pseudoxanthomonas*, *Comamonas*, *Burkholderia*, and *Novosphingobium*), *Firmicutes* (*Bacillus* and *Paenibacillus*), and *Actinobacteria* (*Rhodococcus* and *Arthrobacter*) displayed the ability to degrade various SOCs. Metabolic studies, genomic and metagenomics analyses have aided our understanding of the catabolic complexity and diversity present in these simple life forms which can be further applied for efficient biodegradation. The prolonged persistence of PAHs has led to the evolution of new degradative phenotypes through horizontal gene transfer using genetic elements like plasmids, transposons, phages, genomic islands, and integrative conjugative elements. Systems biology and genetic engineering of either specific isolates or mock community (consortia) might achieve complete, rapid, and efficient bioremediation of these PAHs through synergistic actions. In this review, we highlight various metabolic routes and diversity, genetic makeup and diversity, and cellular responses/adaptations by naphthalene and substituted naphthalene-degrading bacteria. This will provide insights into the ecological aspects of field application and strain optimization for efficient bioremediation.

## Introduction

**FIGURE 1:**
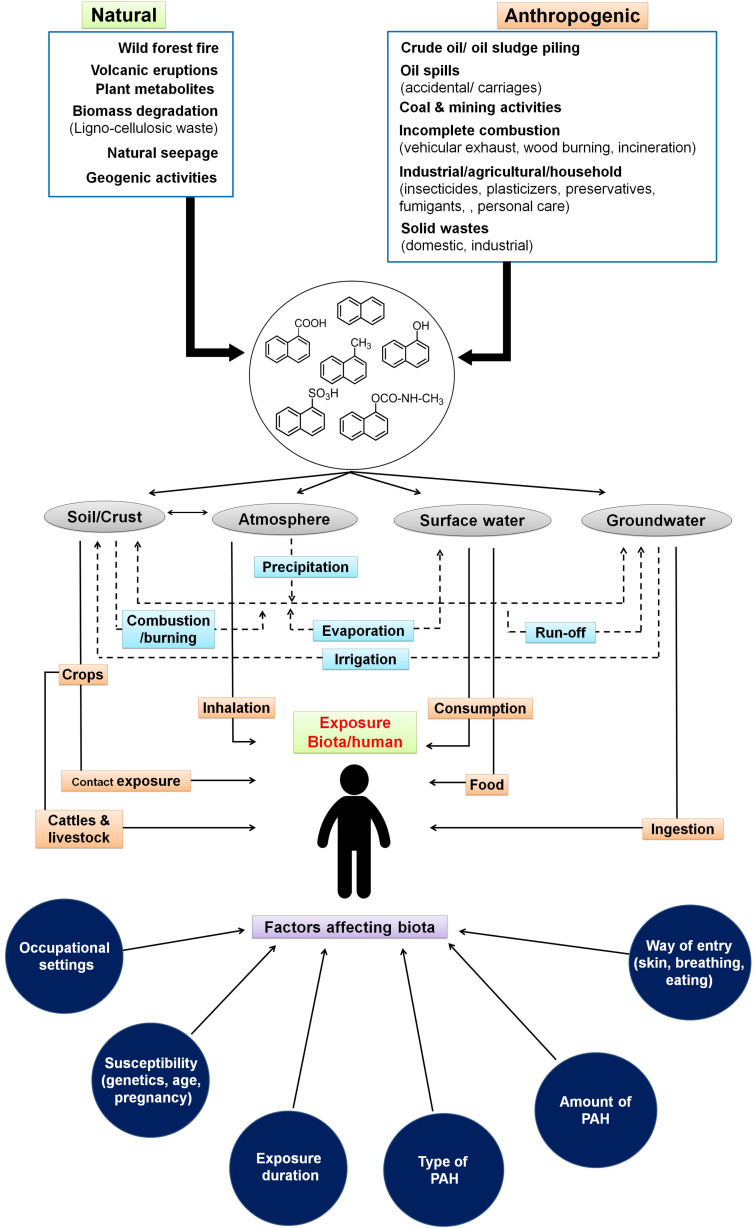
Sources and routes for entry of low molecular weight polycyclic aromatic hydrocarbons through various ecological compartments and various factors affecting biota. Dotted lines indicate the interaction among various compartments of the ecosystem.

The rapid expansion of industries (petrochemical, agricultural, pharmaceutical, textile and dyes, cosmetic, etc.) has led to global economic prosperity and better living standards. This exponential development has resulted in the generation of a huge volume of synthetic organic compounds (SOCs) which are used in the manufacturing of various products. These xenobiotics or SOCs include polycyclic aromatic hydrocarbons (PAHs), pesticides, herbicides, plasticizers, dyes, pharmaceutical products, organophosphates, flame retardants, volatile organic solvents, etc. Their release into atmospheric, aquatic, and terrestrial ecosystems exerts multidimensional effects by altering physicochemical properties and community structure and wielding deleterious effects on various living forms (Petrie et al., 2015; Bernhardt et al., 2017; Sarkar et al., 2020). Many aromatic pollutants have shown critical and deteriorating effects on a number of pristine ecosystems/biodiversity hot spots like coral reefs, Arctic/Antarctic ice caps, high-altitude lakes, deep sea sediment, etc. (Jones, 2010; Beyer et al., 2020; Nordborg et al., 2020). Recent geomicrobiological studies have shown that the deposition of synthetic organics (like aromatic pollutants) and its derivatives on the surface of human-built structures (built environment), i.e., cultural heritages and granitic/stone/wooden/metal monuments, is accelerating its decay (Gadd, 2017; Liu et al., 2018). Human activities can enhance and intensify the biodeterioration of monuments and structures through atmospheric pollution and climate change (Liu et al., 2020). These organic pollutants react with each other in the presence of atmospheric water vapor and get deposited on to the structures leading to physical and chemical deterioration of the material. Biodeterioration has been widely recognized as biologically induced undesirable change in the appearance and properties of a material, affecting its preservation (Pochon and Jaton, 1967). Further microbial influences (metabolism) of these compounds diminish structural integrity, preservation, and cultural importance (Gadd, 2017; Liu et al., 2018). On the other hand, in a few cases, microbial adaptation on these structures and their response were found to be beneficial due to the formation of biofilm and other protective encrustations, lowering the decay/decomposition rate (Martino, 2016). Therefore, effective strategies for the long-term sustainable conservation of stone/metal/wood monuments require an in-depth understanding of the key processes involved. Compared with natural processes (geological processes, wild forest fires, volcanic eruptions, plant and bacterial reactions), anthropogenic activity results in the release of large quantities of PAHs and other SOCs into the ecosystems. Many of the PAHs used in agriculture (insecticides and pesticides like DDT, atrazine, carbaryl, pentachlorophenols, etc.), industries (crude oil, oil sludge/waste, petroleum-derived plastics, polychlorinated biphenyls, plasticizers, detergents, disinfectants, fumigants, fragrances, and preservatives), personal care products (sunscreens, antiseptics, insect repellent, and polycyclic musks), and ammunition (explosives such as 2,4,6-TNT) are potential xenobiotics and impact the planetary health (Srogi, 2007; Vamsee-Krishna and Phale, 2008; Petrie et al., 2015). This list can be further broadened with compounds derived from petroleum products (fuel oil, lubricants, asphaltene), high molecular weight bioplastics, and ionic liquids (Amde et al., 2015). The list of a wide spectrum of aromatic pollutants and its use in various industries is depicted in Table 1. The recent past has also witnessed the onset of ramped-up levels of anthropogenic emissions of volatile organics along with CO_2_ and other greenhouse gases (Dvorak et al., 2017). Nevertheless, the anthropogenic input far exceeds the natural sources. In addition, a spectrum of SOCs is found to be present persistently in many ecological compartments and designated as emerging contaminants which have shown adverse negative effects on living community (Figure 1). Environmental authorities like the United States Environmental Protection Agency (USEPA) have recognized many of them as priority pollutants due to cytotoxic, genotoxic, mutagenic, and carcinogenic activities. Thus, strict guidelines for their disposal and effective strategies for their cleanup/removal from polluted ecosystems are demanded. Various physical and chemical cleanup methods like pyrolysis, oxidative thermal treatment, air-sparging, land-filling, incineration, etc., which are ineffective and expensive, have led to the generation of corrosive, toxic, and recalcitrant by-products. With the increase in global environmental awareness, microbes with the ability to degrade these pollutants and their derivatives (like halo-, nitro-, alkyl-, and/or methyl-) have received an increasing attention (Fennell et al., 2004; Haritash and Kaushik, 2009; Phale et al., 2020; Sarkar et al., 2020; Schwanemann et al., 2020). The use of these indigenous microbial candidates either alone or as mixed culture (consortia) for the removal of aromatic pollutants has been advantageous in terms of environmental safety, cost, efficiency, effectiveness, and sustainability. Researchers are also exploring the combined application of microbiological processes and electrochemical oxido-reduction methods, termed as bioelectrochemical systems (BESs), as a promising technology for pollutant treatment/removal (Huang et al., 2011). Due to its high efficiency, low cost, environmental safety, ambient operating temperatures with biologically compatible materials, and the recovery of by-products of value (e.g., electricity, fuels, and chemicals), BESs are gaining attention (Pant et al., 2012; Nazari et al., 2020). The advent of high-throughput genome sequencing and omics tools/techniques are significantly adding new information on genetic regulation and proteomic and fluxomic responses of several degrading microbes. The combination of such tools with system biology is further expanding our knowledge on the selection and fine-tuning of target catabolic pathways (as metabolic designing) of microbes for effective and efficient biodegradation. In order to develop effective bioremediation strategies by suitable microbial candidate(s), one needs to understand the biochemical potential, metabolic diversity, and genetic makeup as well as the organism’s ecology (auto-/syn-ecology).

**TABLE 1:** Aromatic compounds used in various industries.

Industries	Compounds	Products
Pharmaceuticals	Lincomycin, sulfathiazole	Antibiotics, antiparasitic agents, ionophores
	Amphetamine, bezafibrate, codeine, carbamazepine, diazepam, ephedrine, ibuprofen, fluoxetine, metformin, methandone, propranolol, valsartan, tramadol, morphine, phthalates, phthalate esters, tamoxifen, warfarin	Anti-inflammatory, anticoagulants, hallucinogens, analgesics, antidepressants, lipid regulators, flexible tubings, blood bags, plastic wares
	Estrone, estriol, mestranol, cholesterol	Synthetic estrogens, androgens
Agricultural, household products, various industries and their wastewater	Naphthalene, creosote, mothballs, methyl naphthalene, carbaryl, chlorpyrifos, diethyl phthalate, tri(2-chloroethyl) phosphate, anthracene, 2,6-di-*tert*-butylphenol, 1,2,3-trichloropropane, phenol, dichlorobenzene, acetophenone, asphalt, coal tar	Insecticides, plasticizers, detergents, flame retardants, feedstock, disinfectants, greasing agents, fumigants, fragrances, food preservatives, waste sludge, crude oil
Personal care products	Bisphenol A, 1-benzophenone, methyl naphthalene, methylparaben, triclosan, phthalates	Odor repellents, polycyclic musks, sunscreen agents, fragrances, antiseptics, emulsifier, preservatives
Arms and ammunition	TNT, nitro-aromatics	Explosive, fire retardants

In this review, we attempt to summarize the degradation of simple PAHs like naphthalene and substituted naphthalenes by various bacterial isolates with respect to metabolic pathways and diversity, enzymes involved in the degradation, the genetic makeup/content and diversity, cellular responses, and various bioremediation aspects. Understanding at the biochemical and molecular levels will help in identifying a suitable host strain which can be further genetically engineered for effective and efficient bioremediation of such priority pollutants. This will further aid in developing strategies to formulate site-specific consortia for effective bioremediation.

## Aromatic Compounds: Source, Fate, and Impact on the Biosphere

The preponderance of toxic and hazardous aromatic compounds (satisfying Huckel’s rule of 4*n* + 2 π electrons, *n* = 1, 2, 3, …) is posing an alarming threat to various ecological compartments like air, soil, sediment, and surface and groundwater (Puglisi et al., 2007). With either single- (monocyclic) or multiple-benzene rings (polycyclic) in linear, angular, or cluster arrangement, these compounds have shown stability (persistence/recalcitrance) in the environment due to higher negative resonance energy and inertness (unreactive), which can be attributed to their hydrophobicity and reduced state. Further substitution of aromatic ring(s) by methyl (–CH_3_), carboxyl (–COOH), hydroxyl (–OH), or sulfonate (–HSO_3_) groups renders them more stable, with higher affinity toward macromolecules and bioaccumulation property in the biological systems (Seo et al., 2009; Phale et al., 2020). Several of such low molecular weight polycyclic aromatic hydrocarbons (LMW-PAHs), viz. naphthalene and its derivatives [methyl naphthalenes, naphthoic acids, naphthalene sulfonates and 1-naphthyl *N*-methylcarbamate (carbaryl)], have been considered as priority organic pollutants by the USEPA with genotoxic, mutagenic, and/or carcinogenic effects (Cerniglia, 1984). The release of such LMW-PAHs in the environment results in the bioaccumulation of these compounds in the food chain at various levels, thus affecting the health of the ecosystem (Binkova et al., 2000; Srogi, 2007; Quinn et al., 2009).

The sources and routes of exposure of PAHs to biota primarily occur through transport and interaction among various components of the ecosystem like soil, groundwater, surface water, crops, and atmosphere (Arey and Atkinson, 2003). The interplay and partitioning of various LMW-PAHs in the ecosystem and its exposure routes to biota/human are depicted in Figure 1. Atmospheric contamination and transport (drifting) through vehicular emission, industrial exhaust (coal gasification, combustion, and coke production), and its precipitation leads to PAH deposition on surfaces. Industrial activities like the production of synthetic textiles, dyes, and paint; wood preservation; rubber processing; cement manufacturing activity; pesticide production; and application in agriculture are the main contributors of PAHs in terrestrial and aquatic systems (Bamforth and Singleton, 2005; Wick et al., 2011). It has been shown that soils of peri-urban to urban areas, near highway sites, and larger cities are more exposed to PAHs because of emissions from power plants, residential heating, airway/road traffic burden, and construction activities (Suman et al., 2016). Wang et al. (2008) have shown a maximum of 7,189 μg/kg PAHs in soils close to the roads in comparison to open spaces (2,404 μg/kg) in New Orleans, LA, United States. Similarly, as high as 300 g/kg PAHs have been reported from areas near coal gasification sites in various cities of the United States (Kanaly and Harayama, 2000; Bamforth and Singleton, 2005). Soils from various cities of India like Delhi (Sharma et al., 2008), Agra (Dubey et al., 2014), Mumbai (Kulkarni and Venkataraman, 2000), and Visakhapatnam (Kulkarni et al., 2014) were reported to contain higher concentrations of PAHs. Aromatic compounds have a higher tendency to adsorb onto soil particles, organic matter, and clay minerals, thus acting as a major sink in the ecosystem (Srogi, 2007; Peng et al., 2008). Atmospheric deposition (wet/dry deposition and vapors), runoff from urban sites, wastewater discharges, groundwater recharge, etc. are the major contributors for PAHs into aqueous ecosystems (Srogi, 2007). It has been estimated that around 80% of total PAHs in the marine ecosystem have originated from atmospheric precipitation, deposition, and dumping of waste (Motelay-Massei et al., 2006; Srogi, 2007). The higher levels of PAHs in surface water or leachates from solid waste disposal sites ultimately channel to groundwater, thus creating more vulnerability to the community’s health, as groundwater is consumed by over 70% of populations of Southern and South-Eastern Asia (Duttagupta et al., 2019). A recent study by Duttagupta et al. (2020) involving samples from river (32) and groundwater (235) locations of West Bengal, India, has shown that an estimated 53% of urban and 44% of rural residents (total of 20 million residents) are potentially exposed to naphthalene (4.9–10.6 μg/L) and its derivatives. Differential land-use pattern and increased groundwater pumping/abstraction have been suggested as main controlling factors for vertical transport (advection) of LMW-PAHs in subsurface regimes. River basins and subsurface sediments are found to be impacted by PAHs due to agricultural runoff and domestic and industrial wastewater discharge as well as solid waste/garbage dumping. The loadings are further enhanced through atmospheric precipitation. Higher concentrations of PAHs and its alkyl derivatives (total of 51 types) have been reported from various rivers/river basins across the globe like Fraser, Luanhe, Densu, Missouri, Anacostia, Ebro, Delaware, etc. (Yunker et al., 2002; Motelay-Massei et al., 2006; Li et al., 2010; Amoako et al., 2011; Kim et al., 2018). Naphthalene and phenanthrene are found to be the most predominant (detected in 70% of the samples) in the sediments of the Ganga River basin (Duttagupta et al., 2019). It has been also observed that chlorination of drinking water may lead to the formation of more toxic oxygenated and chlorinated PAHs (Manoli and Samara, 1999). Accumulation of PAHs into grains, fruits, and vegetables occurs through uptake by plants from contaminated soil, groundwater, and atmospheric deposition (Fismes et al., 2002). Many aquatic biota like fish, mussels, shellfish, and shrimp are found to be contaminated with PAHs through ingestion of contaminated food, marine water, and absorption in the tissues and skin (Mackay and Fraser, 2000). Food cooking/processing methods like grilling, barbecuing, smoking, frying, roasting, drying, baking, and charbroiling also contribute a significant amount of PAHs into foods. This is highly dependent on the choice of smoking material, phenolic/aromatic content, cooking procedures, heater type, moisture content, oxygen availability, and combustion temperature (Guillén et al., 2000; Gomes et al., 2013). PAHs have also been detected in milk at varying levels (0.75–2.1 mg/L) (Girelli et al., 2014). Accumulation of these PAHs in food also depends on the physicochemical properties of food, whereas its toxicity effects are linked to the organism’s physiology, metabolic activity, uptake, distribution, and partitioning in the body (Menichini et al., 2011).

The toxicity and the hazardous impact of PAHs were known a long time ago (Cerniglia, 1984). The LMW-PAHs (two to three rings) can bind covalently to various macromolecules like DNA, RNA, and proteins and exert carcinogenicity (Santarelli et al., 2008). Due to their hydrophobic nature, they get partitioned into the lipid membranes. In humans, cytochrome-P_450_ monooxygenase oxidizes PAHs to epoxides, some of which are highly reactive (such as bay-region diol epoxides) and responsible for the transformation of normal cells to malignant ones (Marston et al., 2001). In addition, the PAH transformation products like quinones, phenolics, epoxides, diols, etc. are more toxic than the parent compounds. Several PAHs and their metabolic intermediates have the ability to interfere with hormones and various enzymes in metabolism leading to adverse effects on growth, the central nervous system, and the reproductive and immune systems (Swetha and Phale, 2005; Vamsee-Krishna et al., 2006; Oostingh et al., 2008). Short-term exposure to LMW-PAHs has been reported to cause impaired lung function in asthmatic patients with thrombotic effects and increased risk of skin, lung, bladder, and gastrointestinal cancers (Olsson et al., 2010; Diggs et al., 2011). Animal studies have also shown adverse reproductive and developmental effects from PAH exposure and may further induce cataracts and cause kidney and liver damage and jaundice. Various biotransformation products of PAHs like diols, epoxides, quinones, and free radicals (cations) have shown to form DNA adducts. The stable adducts have shown to alter DNA replication machineries, while unstable adducts depurinate DNA (mostly adenine, but sometimes guanine); both generate errors leading to mutations (Schweigert et al., 2001). In addition, quinones (benzo-/ubi-) can generate reactive oxygen species (ROS) that confer lethal damage to DNA and other macromolecules, thus affecting function/viability of the tissue (Ewa and Danuta, 2017). Long-term exposure to low concentrations of pyrene, biphenyl, and naphthalene has been reported to cause cancer in laboratory animals (Diggs et al., 2012). Owing to their lethal and toxic effects, the cleanup/removal of these PAHs from the impacted/polluted sites is a priority.

Various physical and chemical methods to remove PAHs from contaminated sites/environments have been employed. Processes like incineration, dechlorination, UV oxidation, fixation, solvent extraction, etc. have several drawbacks like toxic by-product formation, complex process, safety and regulatory issues, inefficiency, and high cost. However, microbe-mediated biodegradation, referred to as bioremediation, which involves the application of microbe(s) either as a pure culture or as consortia, is a promising alternative. This process is eco-friendly, non-invasive, cost effective, and sustainable as compared with physical and chemical methods. Bioremediation can be carried out at the impacted site (*in situ*) or in a specially prepared place (*ex situ*) and hence considered as a sustainable cleanup alternative than the conventional physical and chemical methods (Juhasz and Naidu, 2000; Andreoni and Gianfreda, 2007; Megharaj et al., 2011; Phale et al., 2020; Sarkar et al., 2020).

From the ecological and environmental sustainability viewpoint, understanding the microbial metabolic steps involved in the degradation of aromatic pollutants is of the highest scientific and economic value. It has been estimated that a net 2.1 × 10^18^ g carbon (C) has been preserved in the form of sediment rocks and organic compounds, viz. oil, natural gas, and coal (fossil fuels), significantly contributing to the global C cycle. However, rapid industrialization, fossil fuel exploitation, and human activities are exploring such lithospheric carbon pool and adding ∼5.5 × 10^15^ g of organic carbon (in the form of pollutants) to the atmosphere each year (Gonzalez-Gaya et al., 2019). The majority of this organic carbon is contributed to the terrestrial and marine ecosystems through deposition, transport, and runoff. Further, fossil fuel-derived emerging synthetic contaminants, i.e., plastics, plasticizers, and plastic stabilizers (phthalates, its isomers), are predominantly contaminating marine, soil, and water ecosystems and its biota critically, thus contributing to a global climate risk. A range of polyethylene terephthalate (PET)-derived micro- and nano-plastics, plastic debris, and their toxic monomeric products have been assembled together in the Pacific region in between North America and South-East Asia, forming “great Pacific garbage patch” leading to the destruction of marine flora and fauna (Newell et al., 2020). Scientific expeditions have proved the non-feasibility of removing such pollutants/garbage by any physicochemical means. In this context, microbes with oxidative metabolism of pollutants to CO_2_, chemical energy, and other non-toxic by-products, which ultimately get influx to other nutrient cycling processes (H, O, N, S, P, Fe, etc.), are most beneficial. Thus, understanding the microbial ecophysiology of aromatic pollutant mineralization and its ecological controls is critical for estimating microbial C cycling, net C balance, and future climate risks. Considering as high priority the removal of such compounds from the environment, various eco-industries focusing on cleanup technologies are developed. Alternatively, valorization of industrial waste/waste chemicals (i.e., waste-to-wealth approach) accumulating in the ecosystem is recognized to be one of the pillars of the circular economy and sustainable development goal (Close et al., 2012). So, for efficient removal and bioremediation of such aromatic pollutants, it is important to understand the metabolic, enzymatic, and genetic aspects of these potential degrading candidates.

## Naphthalene and Substituted Naphthalenes as Model Compounds

Among several aromatic pollutants, we intended to focus on low molecular weight PAHs like naphthalene and substituted naphthalenes. These compounds are found to be the main components of petroleum-derived fuels, textile dyes, consumer products, pesticides (mothballs and insect repellents), plasticizers, and tanning agents, hence ubiquitously present in many ecosystems (Preuss et al., 2003). Recent reports have highlighted the accumulation of higher concentrations of naphthalene in aquifer sediment, groundwater and subsurface soil, vadose zone, and river beds, signifying its bioaccumulation in the environment (Duttagupta et al., 2019, 2020). The physicochemical properties, applications, and health effects of naphthalene and naphthalene-based derivatives are summarized in Table 2. Compared with other higher molecular weight PAHs, naphthalene and its derivatives are often used as model substrates to study PAH metabolism, genetics, and metabolic diversity as they are less hydrophobic, more soluble in water, and abundant in the ecosystem. A large number of microbes have the ability to metabolize them with comprehensive information on metabolic pathways, enzymes, and regulatory features (Mallick et al., 2011; Phale et al., 2019, 2020). In addition, because of higher abundance and bioavailability, these compounds are designated as prototypic (signature) compounds to assess the pollution in the environment. The USEPA estimated an average of 5.19 μg naphthalene/m^3^ derived primarily from the incomplete combustion of fuels, 0.3–4 μg from cigarette smoke, 7.8–46 μg from sidestream smoke, and 100- to 10,000-fold higher exposure through creosote and mothball manufacturing (Preuss et al., 2003). Particularly, naphthalene is found to have species-, regional-, and sex-selective respiratory toxicity and carcinogenic effects. Based on evidences from animal studies, the International Agency for Research on Cancer (IARC) has classified naphthalene to be a “likely human carcinogen” (group 2B)^1^. Exposure to substituted naphthalenes is predominantly through inhalation or parenteral administration (oral consumption) leading to damage of lung tissues with increased incidences of pulmonary tumors in rats and mice (National Toxicology Program^2^). Nausea, vomiting, abdominal pain, diarrhea, headache, confusion, profuse sweating, fever, tachycardia, etc. are the consequences of acute exposure. On the other hand, carbaryl (1-naphthyl *N*-methylcarbamate), a broad-spectrum carbamate insecticide, has been reported to be toxic to aquatic invertebrates, amphibians, bees, and humans and has been shown to inhibit acetylcholine esterase leading to paralysis (Smulders et al., 2003; Bulen and Distel, 2011). Thus, it is imperative to understand the microbial degradation mechanisms, genetic regulations, enzymes, and cellular response to strategize its bioremediation from a contaminated environment.

**TABLE 2:** Details of the physicochemical properties of naphthalene and its derivatives, its application, identification methods, and associated diseases.

**Structure and physicochemical properties**	**Naphthalene**	**1-Methyl naphthalene**	**2-Methyl naphthalene**	**1-Naphthoic acid**	**1-Naphthyl *N*-methylcarbamate (Carbaryl)**	**Naphthalene monosulfonate**	**Naphthalene disulfonate**
	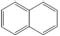	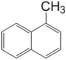	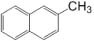	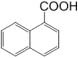	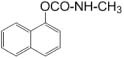	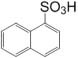	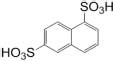
Mol. wt. (Da)	128.17	142.2	142.2	172.18	201.22	207.23	202.23
Density (mmHg at 77°F)	1.15	1.01	1.01	1.3	1.23	0.38	0.4
Vapor pressure	0.05	23	0.06	0.06	1.36 × 10^–6^	7.6 × 10^–8^	7.5 × 10^–8^
O/W coefficient (Log*P*)	3.3	3.87	3.86	2.54	2.36	−1.78	−1.85
Autoignition temperature (°)	979	984	–	–	Not flammable	Not flammable	Not flammable
Chemical safety	Irritant, health hazard	Irritant, health hazard	Irritant	Irritant	Irritant, health hazard	Irritant	Irritant
Toxic/lethal dose	5–15 g	0.03–0.5 ml	–	–	500 mg	1,390 mg	2,300 mg
Applications	Insecticide repellent Fuels-additives Lubricants Paint Abrasives Adhesives	Adjuvant Scrubber oil Feedstock Beverages	Insecticide Dye carrier Feedstock Textile surfactants	Pesticide Metabolite-intermediate	Pesticide Fungicide Growth regulator Cleaning agent Cosmetics	Dye carrier Dry-cleaning agent Degreaser	Dye carrier Degreaser Liposome preparation
Identification methods	GC-MS	GC-MS RP-HPLC	GC-MS	GC-MS	RP-HPLC	HPLC	HPLC
Associated diseases	Kidney damage Anemia Cataract Hyperplasia Hepatomegaly	Skin–eye irritation Respiratory depression	Eye irritation Respiratory congestion Pyretic	Respiratory congestion Behavioral convulsion	Asthma, bronchitis Agranulocytosis Catalepsy Melanoma	Dermal rashes Corneal damage Kidney malfunction	Dermal rashes Corneal damage Kidney malfunction

## Microbial Degradation of Naphthalene and Substituted Naphthalenes

### Taxonomic Diversity of Degrading Microbes

In contaminated niches, hydrophobic and lipophilic aromatic pollutants exert varied cellular effects on environmental microbiome (communities), e.g., altering the membrane fluidity, permeabilization of membranes, swelling of lipid bilayers, disruption in energy transduction (electron transport chain/proton motive force), and the activity of membrane-associated proteins (Sikkema et al., 1995). Additionally, some soluble intermediates like catechols and quinones produce ROS and form adducts with DNA and proteins (Penning et al., 1999). Thus, the abundance of such compounds in the ecosystem acts as a selection pressure on the microbial community to evolve as an efficient degrader at various physiological levels such as uptake/transport, intracellular transformation, assimilation/utilization, and compartmentalization.

**FIGURE 2:**
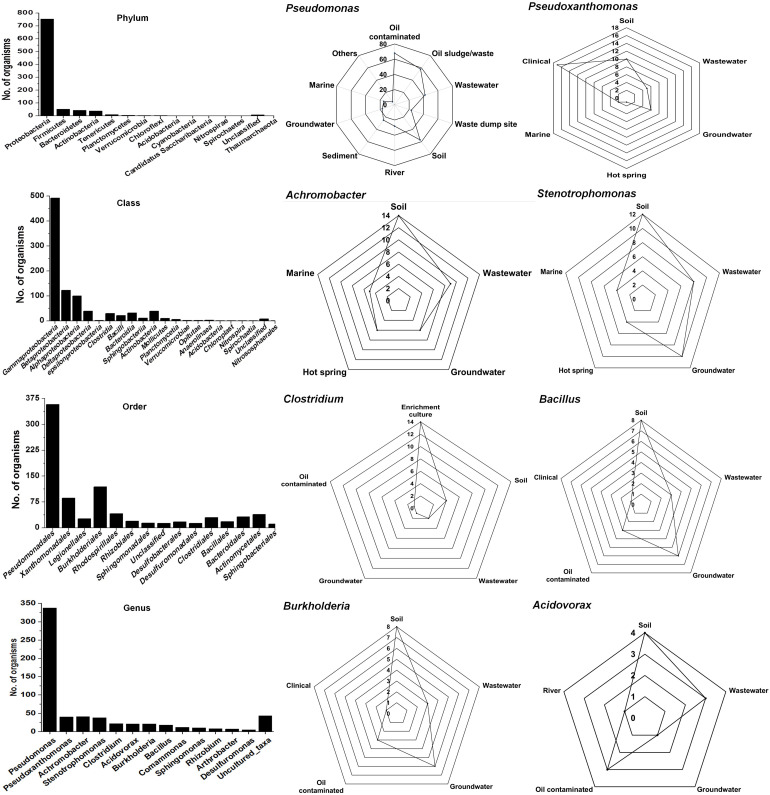
Taxonomic diversity and ecological distribution of bacterial members from the environment contaminated with naphthalene and naphthalene derivatives.

The Ribosomal Database Project-II (RDP-II) search shows that a total of 926 bacteria have been isolated from the environment contaminated with naphthalene or its derivatives or enrichment cultures. *Proteobacteria* members represented the maximum (*n* = 755), followed by *Firmicutes* (52), *Bacteroidetes* (43), *Actinobacteria* (39), *Tenericutes* (10), and unclassified bacteria (8) (Figure 2). Members of γ*-proteobacteria* (*Pseudomonadales* and *Xanthomonadales*) are predominantly (54%) reported among all Gram-negative, high G + C content groups, whereas *Clostridiales* and *Bacillales* (30%) are among the Gram-positive low G + C content groups. *Pseudomonas* (a total of 338 spp., being the highest) are reported for the degradation of naphthalene and its methyl derivatives from many contaminated (coal tar, petroleum, crude oil, oil sludge, spillage, wastewater, organic waste, and dumpsites) and pristine (soil, river, sediment, and groundwater) ecosystems (Figure 2). Besides, enrichment studies and metagenomic analysis of some of these sites have indicated the probable degradation capacity of uncultivable *Legionella* and *Clostridium* members, indicating the need of cultivating these bacteria to understand novel pathways and metabolic diversity.

### Metabolic Pathways for Complete Utilization/Mineralization

#### Naphthalene

**FIGURE 3:**
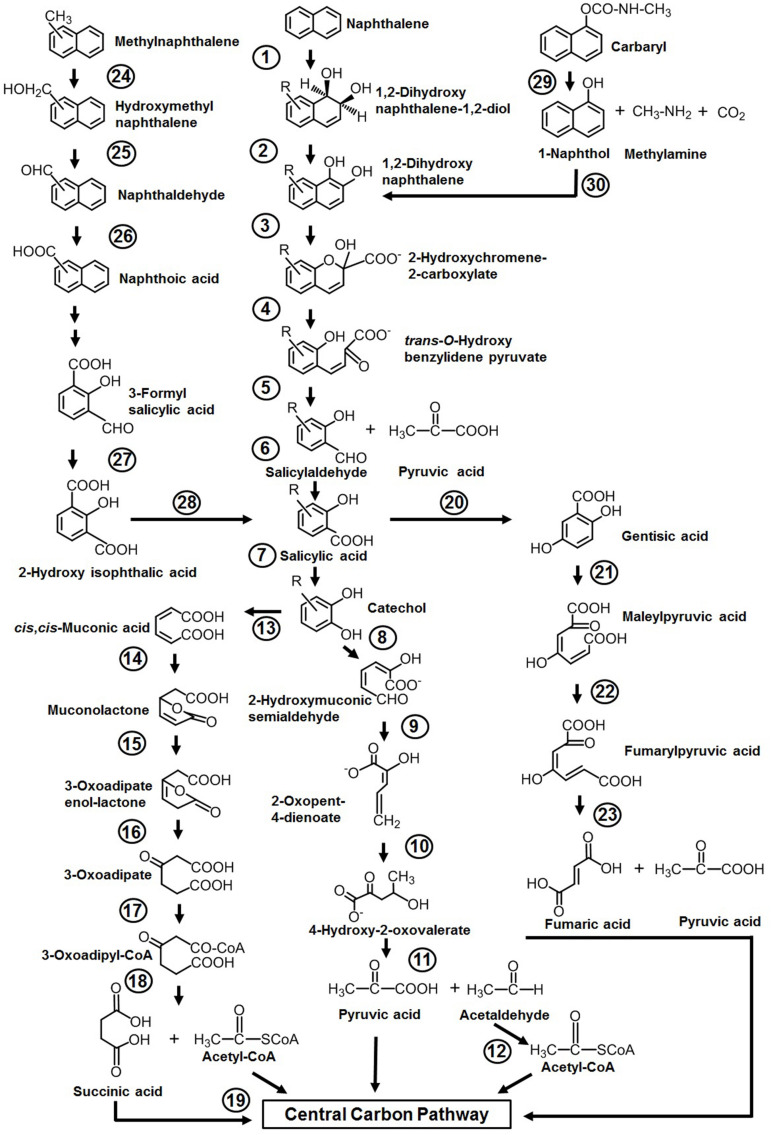
Pathways for the degradation of naphthalene, methylnaphthalenes, naphthoic acid, and Carbaryl. Numbers within circles represent enzymes responsible for sequential conversion of naphthalene and its derivatives to its subsequent products. 1, Naphthalene dioxygenase (NDO); 2, *cis*-dihydrodiol dehydrogenase; 3, 1,2-dihydroxynaphthalene dioxygenase; 4, 2-hydroxychromene-2-carboxylate isomerase; 5, *trans-O*-hydroxybenzylidene pyruvate hydratase-aldolase; 6, salicylaldehyde dehydrogenase; 7, salicylate 1-hydroxylase; 8, catechol 2,3-dioxygenase (C23DO); 9, 2-hydroxymuconic semialdehyde dehydrogenase; 10, 2-oxopent-4-enoate hydratase; 11, 4-hydroxy-2-oxovalerate aldolase; 12, acetaldehyde dehydrogenase; 13, catechol 1,2-dioxygenase (C12DO); 14, muconate cycloisomerase; 15, muconolactone delta-isomerase; 16, β-ketoadipate enol-lactone hydrolase; 17, β-ketoadipate succinyl-CoA transferase; 18, β-ketoadipyl-CoA thiolase; 19, succinyl-CoA:acetyl-CoA succinyl transferase; 20, salicylate 5-hydroxylase; 21, gentisate 1,2-dioxygenase (GDO); 22, maleylpyruvate isomerase; 23, fumarylpyruvate hydrolase; 24, methylnaphthalene hydroxylase (NDO); 25, hydroxymethylnaphthalene dehydrogenase; 26, naphthaldehyde dehydrogenase; 27, 3-formylsalicylate oxidase; 28, hydroxyisophthalate decarboxylase; 29, carbaryl hydrolase (CH); 30, 1-naphthol 2-hydroxylase.

Among various reported microbes for the degradation of aromatics, the majority of them have the ability to degrade naphthalene as the sole source of carbon and energy. The sequence of events involved in naphthalene metabolism has been reported from *Pseudomonas putida* (strains: NCIB 9816-4, G7, AK-5, PMD-1, and CSV86), *Pseudomonas stutzeri* AN10, *Pseudomonas fluorescens* PC20, and other spp. (ND6 and AS1) (Mahajan et al., 1994; Resnick et al., 1996; Annweiler et al., 2000; Basu et al., 2003; Dennis and Zylstra, 2004; Sota et al., 2006; Izmalkova et al., 2013). The metabolism is initiated by a multicomponent dioxygenase enzyme [naphthalene dioxygenase (NDO), ring-hydroxylating dioxygenase] which catalyzes the oxidation of one of the aromatic rings of naphthalene using molecular oxygen as another substrate to convert naphthalene to *cis*-naphthalene dihydrodiol (Figure 3). *cis*-Dihydrodiol is converted to 1,2-dihydroxynaphthalene by dehydrogenase. Ring-cleaving dioxygenase, 1,2-dihydroxynaphthalene dioxygenase (12DHNDO), converts 1,2-dihydroxynaphthalene to 2-hydroxychromene-2-carboxylic acid. An enzymatic *cis*–*trans* isomerization forms *trans-o*-hydroxybenzylidene pyruvate, which gets cleaved by a hydratase-aldolase to salicylaldehyde and pyruvate. The organic acid pyruvate is the first C3 compound derived from naphthalene carbon skeleton and channelized to central carbon pathway. Further, NAD^+^-dependent salicylaldehyde dehydrogenase converts salicylaldehyde to salicylate. Metabolism up to this step is referred to as the “upper pathway” for naphthalene degradation. This route is very common in most of the naphthalene degraders. However, there are few exceptions; for example, in *Bacillus thermoleovorans* Hamburg 2, naphthalene degradation is initiated by naphthalene 2,3-dioxygenase to yield 2,3-dihydroxy-naphthalene (Annweiler et al., 2000).

Depending on the organism and its genetic makeup, the generated salicylate is further metabolized either *via* the catechol route using salicylate 1-hydroxylase (S1H) or the gentisate route employing salicylate 5-hydroxyalse (S5H) (Figure 3). As salicylate is designated to be a major intermediate of naphthalene metabolism (upper pathway), the steps from salicylate to TCA intermediates often termed as lower pathway and genes are organized as a single operon. It is often seen that both the upper pathway (*nah*) operon and lower pathway (*sal* operon) genes are regulated through a common regulator; for example, NahR and salicylate act as an inducer for both operons to metabolize naphthalene completely (Phale et al., 2019, 2020).

Further, catechol is ring-cleaved through the *meta* route by catechol 2,3-dioxygenase (C23DO) to 2-hydroxymuconic semialdehyde (Yen et al., 1988), which is further hydrolyzed by 2-hydroxymuconic semialdehyde hydrolase to yield 2-hydroxypenta-2,4-dienoate. Subsequent actions of hydratase (2-oxopent-4-enoate hydratase) and aldolase (4-hydroxy-2-oxovalerate aldolase) convert 2-hydroxypenta-2,4-dienoate into pyruvic acid and acetaldehyde which are then funneled into the central carbon pathway (Figure 3). Alternatively, catechol is ring-cleaved *via* the *ortho* route by catechol 1,2-oxygenase (C12DO) to yield *cis,cis*-muconic acid. Muconate cycloisomerase, muconolactone isomerase, and β-ketoadipate enol-lactone hydrolase convert *cis,cis*-muconic acid to 3-oxoadipate which enters central carbon pathways *via* succinyl-CoA and acetyl-CoA (Nozaki et al., 1968) (Figure 3).

In the gentisate (2,5-dihydroxybenzoate) pathway, the aromatic ring is cleaved by gentisate 1,2-dioxygenase (GDO) to produce maleylpyruvate. This product can directly hydrolyze to pyruvate and malate or may undergo into isomerization to yield fumarylpyruvate which is hydrolyzed to pyruvate and fumarate (Larkin and Day, 1986). At the biochemical and genetic levels, alternative pathway selection has been noticed in Gram-negative and Gram-positive bacteria (Morawski et al., 1997; Whyte et al., 1997). Gram-negative members (*Pseudomonas*) prefer salicylate for decarboxylation by salicylate 1-hydroxylase to yield catechol, where salicylate acts as an inducer for naphthalene metabolism (Gibson and Subramanian, 1984). On the other hand, in Gram-positive bacteria (*Rhodococcus*), salicylate 5-hydroxylase is employed to convert salicylate to gentisate, where salicylate has no induction effects on the transcription of naphthalene genes (Grund et al., 1992) (Figure 3).

#### Methylnaphthalenes

Organisms like *P. putida* CSV86, *Marinobacter* sp. NCE312, *Neptunomonas naphthovorans*, *Sphingomonas paucimobilis* 2322, *Vibrio cyclotrophicus*, *P. fluorescens* LP6a, *Pseudomonas* spp., and *Mycobacterium* have been reported to degrade mono- or di-methylnaphthalenes (Dean-Raymond and Bartha, 1975; Cane and Williams, 1982; Mahajan et al., 1994; Dutta et al., 1998; Hedlund et al., 1999). Among these, the degradation pathway for 1- and 2-methylnaphthalene from *P. putida* CSV86 has been well elucidated at the biochemical and enzymatic levels (Mahajan et al., 1994). 1-Methylnaphthalene is metabolized by two routes, firstly by aromatic ring-hydroxylation (of the unsubstituted ring of methylnaphthalene) to yield *cis*-1,2-dihydroxy-1,2-dihydro-8-methylnaphthalene, which is further oxidized to methylsalicylate and methylcatechol, which upon ring-cleavage channeled to the central carbon pathway (Figure 3). This pathway is referred to as the “carbon source pathway.” In the second, “detoxification pathway,” the methyl group is hydroxylated, probably by NDO, to form 1-hydroxy methylnaphthalene which is further oxidized to 1-naphthoic acid and excreted into the medium as a dead-end product. It has been shown that strain CSV86 failed to grow on 1- and 2-naphthoic acid as the sole source of carbon and energy, confirming the detoxification pathway (Mahajan et al., 1994; Basu et al., 2003). In case of 2-methylnaphthalene, the methyl group is hydroxylated by hydroxylase, resulting in the formation of 2-hydroxymethyl naphthalene. Further, it undergoes ring-hydroxylation of unsubstituted ring to yield dihydrodiol, which gets oxidized by a series of enzyme-catalyzed reactions to form 4-hydroxymethyl catechol and follows the *meta* ring-cleavage route to enter the central carbon pathway. Similarly, *S. paucimobilis* 2322 has been reported to hydroxylate 2-methyl naphthalene by employing NDO, and further oxidation yields methylsalicylate and methylcatechol (Dutta et al., 1998).

#### Naphthoic Acid

Naphthoic acids (substituted/unsubstituted) are formed as detoxification/biotransformation by-products during degradation of methylnaphthalenes, phenanthrene, and anthracene and are excreted into the spent medium. The soil isolate *Stenotrophomonas maltophilia* CSV89 has been reported to metabolize 1-naphthoic acid as a carbon source (Phale et al., 1995). The metabolism is initiated by double hydroxylation of the aromatic ring to yield 1,2-dihydroxy-8-carboxynaphthalene. The resultant diol gets oxidized *via* 2-hydroxy-3-carboxybenzal pyruvate, 3-formylsalicylate, 2-hydroxyisophthalate, and salicylate to catechol and enters *via* the *meta* ring-cleavage route to the central carbon pathway (Figure 3).

#### 1-Naphthyl *N*-methylcarbamate (Carbaryl)

Carbaryl is a naphthalene-based carbamate pesticide. With the onset of the Green Revolution in the 1970s in India, the use of chemical fertilizers and pesticides has increased the PAH load through non-point source agricultural outflow (Pingali, 2012; Duttagupta et al., 2020). An estimated 55% (85,722,000 hectares) of total cultivated farmland are under the use of chemical pesticides. In the last 5 years (2015–2020, till date), an average of 55,000–60,000 metric tons of pesticides/year are used in the Indian agricultural field (Department of Agriculture, cooperation, farmers welfare, Govt. of India^3^, August 2020). The crops grown over the northern and middle Gangetic plain areas (states with the highest population and density) are pervasively using pesticides, among which insecticides are predominant. Carbaryl (1-naphthyl *N*-methylcarbamate) is a wide-spectrum, moderate to very toxic carbamate insecticide with an average use of 100–110 metric tons in Indian agriculture. It is generally sold under the trade name Sevin for controlling insects (aphids, fire ants, fleas, ticks, spiders, and many other outdoor pests) infesting various crops (corn, soybean, cotton, fruits, and vegetables). Few microbes like *Pseudomonas* spp. (NCIB 12042, 12043, C4, C5, C6, C7, *P. putida* XWY-1), *Rhodococcus* sp. (NCIB 12038), *Sphingobium qiguonii* (CF06), *Burkholderia* sp. (C3), *Micrococcus*, and *Arthrobacter* sp. (RC100) are reported to degrade carbaryl (Larkin and Day, 1986; Chapalamadugu and Chaudhry, 1991; Hayatsu et al., 1999; Swetha and Phale, 2005; Trivedi et al., 2017). The carbaryl degradation pathway is studied in detail at the biochemical and enzymatic as well as at the genetic level from the soil isolates *Pseudomonas* sp. strains C4, C5, and C6 (Swetha and Phale, 2005; Trivedi et al., 2016) (Figure 3). The metabolic pathway starts with the hydrolysis of ester bond by carbaryl hydrolase (CH) to form 1-naphthol, methylamine, and CO_2_. The subsequent action of 1-naphthol hydroxylase (1-NH) converts 1-naphthol to 1,2-dihydroxynaphthalene, which is further metabolized to the central carbon pathway *via* salicylic acid and gentisic acid. Few carbaryl degraders were also reported to metabolize it through salicylic acid *via* the catechol *ortho* ring-cleavage route (Larkin and Day, 1986; Chapalamadugu and Chaudhry, 1991). It is interesting to note that, in naphthalene degraders, salicylic acid is predominantly metabolized through catechol, whereas Carbaryl degraders prefer the gentisic acid route for salicylate metabolism.

#### Naphthalene Sulfonates

**FIGURE 4:**
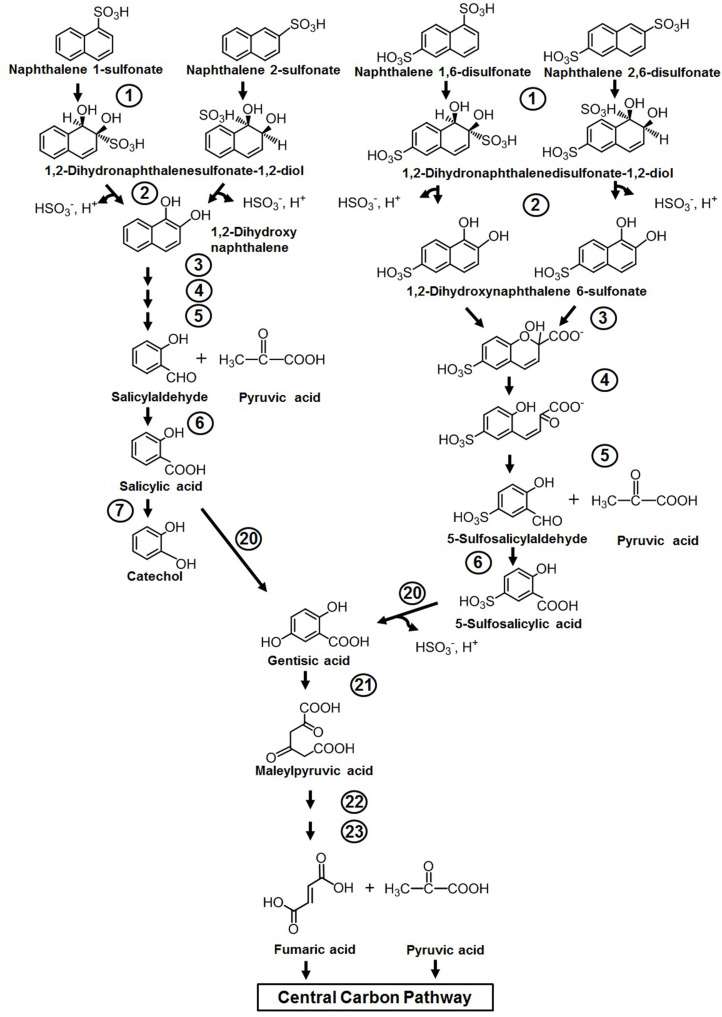
Metabolic pathway for the degradation of naphthalene sulfonates. Numbers within circles represent enzymes responsible for the metabolism of naphthalene sulfonates and are similar/the same as described in Figure 3.

Naphthalene sulfonic/disulfonic acids and naphthylaminesulfonic acid derivatives are used as intermediates in the production of azo dyes, wetting agents, dispersants, etc. Although the compounds are less toxic to humans, cytotoxicity assessment has shown its lethal effects on fish, water fleas (*Daphnia*), and water algae (Greim et al., 1994). Members of *Pseudomonas* spp. (strains A3, C22) have been reported to initiate metabolism through double hydroxylation of the aromatic ring bearing sulfonate group to yield dihydro diol, which is further converted to 1,2-dihydroxynaphthalene by spontaneous elimination of the sulfite group (Brilon et al., 1981). The generated 1,2-dihydroxynaphthalene is catabolized through the classical naphthalene pathway either *via* the catechol or gentisate route (Figure 4). It has also been demonstrated that amino- and hydroxy-naphthalene sulfonic acids are completely degraded by mixed bacterial consortium harboring complementary catabolic pathways (Nortemann et al., 1986). One member of this community was shown to desulfonate amino- or hydroxy-naphthalene sulfonic acids by 1,2-dioxygenation, and amino- or hydroxysalicylates were excreted as dead-end metabolites into the medium, which are subsequently assimilated by other members of the consortium. Naphthalene disulfonic acids, being comparatively more polar but resistant for biodegradation, are metabolized by different pathways. The first desulfonation occurs by regioselective dihydroxylation of the aromatic ring with the sulfonate group, while the second one occurs during hydroxylation of 5-sulfosalicylic acid by salicylate 5-hydroxylase to generate gentisic acid, which enters the central carbon pathway (Brilon et al., 1981) (Figure 4). The enzymes responsible for naphthalene degradation are also responsible for naphthalene sulfonate metabolism (Brilon et al., 1981; Keck et al., 2006).

### Enzymes

The LMW-PAHs are reducing, hydrophobic, and sparingly soluble, thus resistant to natural attenuation/degradation. However, aerobic microorganisms have the ability to oxidize them by incorporating molecular oxygen (O_2_). These enzymes mainly belong to oxido-reductase class and perform various reactions like aromatic ring-hydroxylation (mono- or di-), dehydrogenation, and aromatic ring-cleavage. These reactions yield products with increased oxidation status and are more susceptible for further metabolism to the central carbon pathway (Phale et al., 2020). The degradation pathway enzymes are reported to be inducible. The activities of these enzymes were found to be very low or negligible when cells are grown on a simple carbon source like glucose or organic acids. Various enzymes (oxygenase, hydrolase, dehydrogenase, oxidase, etc.) involved in the metabolism of naphthalene and its derivatives are summarized in Table 3.

**TABLE 3:** Biochemical details of the enzymes responsible for degradation of naphthalene and its derivatives.

Enzymes	Genes	Mol. wt. (in kDa)* and organization^$^	Co-factors	Gene Ontology function
Naphthalene 1,2-dioxygenase reductase (ferredoxin reductase) EC: 1.14.12.12	*nah*Aa	35.5 Trimer	2Fe-2S FAD FMN NADH	Two iron, two sulfur cluster binding Dioxygenase activity Electron transfer activity Metal ion binding
Naphthalene 1,2-dioxygenase (ferredoxin component) EC: 1.14.12.12	*nah*Ab	11.4 Trimer	2Fe-2S NADH	Two iron, two sulfur cluster binding Dioxygenase activity
Naphthalene 1,2-dioxygenase (large subunit) EC: 1.14.12.12	*nah*Ac	55.0 Dimer	2Fe-2S Fe^2+^ NADH	Two iron, two sulfur cluster binding Dioxygenase activity Ion binding Cellular metabolic process
Naphthalene 1,2-dioxygenase (small subunit) EC: 1.14.12.12	*nah*Ad	20.0 Dimer	Fe-S NADH	Dioxygenase activity Cellular metabolic process
*cis*-Dihydrodiol dehydrogenase EC: 1.3.1.29	*nah*B	27.5 Tetramer	NADP^+^ NAD^+^	Cellular metabolic process
1,2-Dihydroxynaphthalene dioxygenase EC: 1.13.11.56	*nah*C	33.9 Monomer	Fe^2+^	Dioxygenase activity Cellular metabolic process
2-Hydroxychromene-2-carboxylate isomerase EC: 5.99.1.4	*nah*D	23.1 Dimer	Glutathione	Isomerase Protein disulfide oxido-reductase Metabolic process
*Trans-O*-hydroxybenzylidene pyruvate hydratase-aldolase EC: 4.1.2.45	*nah*E	36.9 Monomer	–	Aldehyde-lyase activity Hydratase-aldolase activity
Salicylaldehyde dehydrogenase EC: 1.2.1.65	*nah*F	52.0 Monomer	NAD^+^	Dehydrogenase activity Cellular metabolic process
Salicylate hydroxylase EC: 1.14.13.1	*nah*G	46.83 Monomer	NADH	FAD binding Monooxygenase Metabolic process
Catechol 2,3-dioxygenase EC: 1.13.11.2	*nah*H (*xyl*E)	35.2 Tetramer	Fe^2+^	Dioxygenase Ferrous ion binding
2-Hydroxymuconic semialdehyde dehydrogenase EC: 1.2.1.85	*nah*I (*xyl*G)	51.8 Dimer	NAD^+^	Hydroxylation Oxido-reductase
2-Hydroxymuconic semialdehyde hydrolase EC: 3.7.1.9	*nah*N (*xyl*F)	30.6 Monomer	–	Hydrolase
2-Oxopent-4-enoate hydratase EC: 4.2.1.80	*nah*L (*xyl*J)	23.9 Dimer	–	Hydrolase
4-Hydroxy-2-oxovalerate aldolase EC: 4.1.3.39	*nah*M (*xyl*K)	37.4 –	Mn^2+^	Aldolase Metal ion binding
Acetaldehyde dehydrogenase EC: 1.2.1.10	*nah*O (*dmp*F	33.1 Dimer	CoA NAD^+^	Dehydrogenase activity NAD binding
4-Oxalocrotonate decarboxylase EC: 4.1.1.77	*nah*K (*xyl*L)	27.456 Dimer	NAD^+^	Catalytic activity Metal ion binding
4-Oxalocrotonate tautomerase EC: 5.3.2.6	*nah*J (*xyl*H)	6.8 Hexamer	–	Isomerase Metabolic processes
Catechol 1,2-dioxygenase EC: 1.13.11.1	*cat*A	34.2 Dimer	Fe^3+^	Oxido-reductase Metal binding
Muconate cycloisomerase EC: 5.5.1.1	*cat*B	41.1 Octamer	Mn^2+^	Cycloisomerase Metabolic process Metal binding
Muconolactone Delta-isomerase EC: 5.3.3.4	*cat*C	10.6 Decamer	–	Delta-isomerase Metabolic process
β-Ketoadipate enol-lactone hydrolase EC: 3.1.1.24	*pca*D	28.6 Trimer	–	Enol-lactonase
β-Ketoadipate succinyl-CoA transferase (α subunit) EC: 2.8.3.6	*pca*I	24.4 Dimer	–	CoA transferase (binding) Metabolic process
β-Ketoadipate succinyl-CoA transferase (β subunit) EC: 2.8.3.6	*pca*J	22.5 Dimer	–	CoA transferase (binding) Metabolic process
β-Ketoadipyl-CoA thiolase EC: 2.3.1.174	*pca*F (*paa*J)	42.27 Tetramer	–	Thiolase Metabolic process Catalytic activity DNA damage stimulus
Salicylate-5-hydroxylase (large subunit) EC: 1.14.13.172	*nag*G	48.8 Monomer	2Fe-2S Fe^2+^ NAD^+^	Oxido-reductase Metabolic process Metal binding
Salicylate-5-hydroxylase (small subunit) EC: 1.14.13.172	*nag*H	18.8 Monomer	NADH	Oxido-reductase Metabolic process Metal binding
Gentisate 1,2-dioxygenase EC: 1.13.11.4	*nag*I	39.7 –	Fe^2+^	Dioxygenase Metal binding
Maleylpyruvate isomerase EC: 5.2.1.4	*nag*L	23.5 –	Glutathione	Isomerase Amino acid metabolic process
Fumarylpyruvate hydrolase EC: 3.7.1.20	*nag*K	20.9 –	Mg^2+^ Mn^2+^	Hydrolase Metal ion binding
Carbaryl hydrolase EC: 3.1.1.aj	*mcb*A (CH)	85.4 Monomer	–	Integral membrane component
1-Naphthol 2-hydroxylase EC: 1.14.13.M78	*mcb*C (1-NH)	64.7 Dimer	FAD NADPH	Cellular metabolic process
Formylsalicylate oxidase EC: 1.2.3.-	–	– Monomer	–	Cellular metabolic process

*Molecular weight deduced from the nucleotide sequence of the gene. ^$^Determined experimentally.

Radio-isotopic studies (^18^O_2_) have demonstrated that incorporation of molecular O_2_ by oxygenases to the aromatic ring is the most crucial step in activating the compound for further biodegradation (Hayaishi et al., 1955; Mason et al., 1955). Incorporation of a single oxygen atom (O) from the molecular oxygen (O_2_) into the substrate is initiated by either internal or external monooxygenase (also referred to as hydroxylase). The other oxygen atom is reduced to water. External mono-oxygenases use NADH or NADPH to reduce flavin, while in internal mono-oxygenases, flavin is reduced by the substrate. The position of hydroxylation results in the diversity of product formation. For example, salicylate 1-hydroxylase hydroxylates salicylate at C1 position to form catechol. On the other hand, multicomponent salicylate 5-hydroxylase (with reductase, ferredoxin, and oxygenase subunit) performs hydroxylation at the C5 position of salicylate to yield gentisate (Yamamoto et al., 1965).

Dioxygenases incorporate both atoms of O_2_ into the substrate. Based on the product formed, they are grouped either as ring-hydroxylating or ring-cleaving dioxygenase. The ring-hydroxylating dioxygenase converts aromatic substrate to yield *cis*-dihydrodiols (like naphthalene) and is found to be prevalent in bacteria. To date, ring-hydroxylating dioxygenase-containing organisms have been shown to grow on a range of aromatic carbon sources, and these enzymes have been classified as NDO (for naphthalene), toluene dioxygenase (TDO for toluene), and biphenyl dioxygenase (BPDO for biphenyl). Both NDO and BPDO are capable of catalyzing dioxygenation and side-chain hydroxylation of various PAHs (toluene, nitrotoluene, xylene, ethylbenzene, naphthalene, biphenyl, fluorene, indole, methylnaphthalene, naphthalene sulfonates, phenanthrene, anthracene, acetophenones, etc.) (Boyd and Sheldrake, 1998; Phale et al., 2020). The NDO is a multicomponent system that includes oxido-reductase, a ferredoxin, and an oxygenase component containing the active site (Gibson and Subramanian, 1984; Resnick et al., 1996). The catalytic unit of the NDO is composed of large and small subunits (α and β), respectively, arranged in α3β3 configuration. NDO is a member of a large family of oxygenases with α subunits containing a Rieske [2Fe-2S] center and mononuclear non-heme iron, which determines the substrate specificity of NDO (Parales et al., 1998). Generally, two electrons from the reduced pyridine nucleotide are transferred *via* reductase, ferredoxin, and Rieske center to Fe(II) ion at the active site during a catalytic cycle. The reducing equivalents allow the activation of molecular oxygen, which is a prerequisite to dihydroxylation of the substrate (Ferraro et al., 2005). So far, few NDOs have been purified and extensively characterized from different strains of bacteria, and the genetic control of the pathways involved in naphthalene degradation has been studied in detail (Resnick et al., 1996; Parales et al., 1998; Karlsson et al., 2003). The ring-cleaving dioxygenases (intradiol or *ortho* ring-cleavage and extradiol or *meta* ring-cleavage) act on hydroxylated aromatic compounds. For example, the *ortho* ring-cleaving dioxygenase is catechol 1,2-dioxygenase, while the *meta* ring-cleaving is catechol 2,3-dioxygenase (Kojima et al., 1961; Nozaki et al., 1968). Besides various oxygenases, various dehydrogenases are responsible for dehydrogenation of aromatic dihydrodiols, alcohols, and aldehydes and use NAD^+^/NADP^+^ as an electron acceptor and are one of the important enzymes involved in metabolism (Gibson and Subramanian, 1984; Shaw and Harayama, 1990; Phale et al., 2020).

Enzymes like hydrolases (esterases, amidases) are the second important classes of enzymes that utilize water to cleave the covalent bond and display a broad substrate specificity. Carbaryl hydrolase and other hydrolases are proposed to be periplasmic in Gram-negative members as an integral (transmembrane) component (Kamini et al., 2018). Carbaryl possesses an amide as well as an ester linkage; hence, hydrolysis can be catalyzed either by esterase or amidase to yield 1-naphthol. The CH from *Rhizobium* sp. strain AC10023 and *Arthrobacter* sp. strain RC100 was reported to act as esterase and amidase, respectively. CH from *Arthrobacter* sp. RC100 has shown hydrolysis of four *N*-methylcarbamate insecticides, e.g., Carbaryl, xylylcarb, metolcarb, and XMC (Hayatsu et al., 2001). CH from *Pseudomonas* sp. C5pp was reported to act on Carbaryl (100% activity) and 1-naphthylacetate (36%) but not on 1-naphthalene acetamide, suggesting it to be an esterase (Trivedi et al., 2016).

### Genetic Organization and Regulatory Features

**FIGURE 5:**
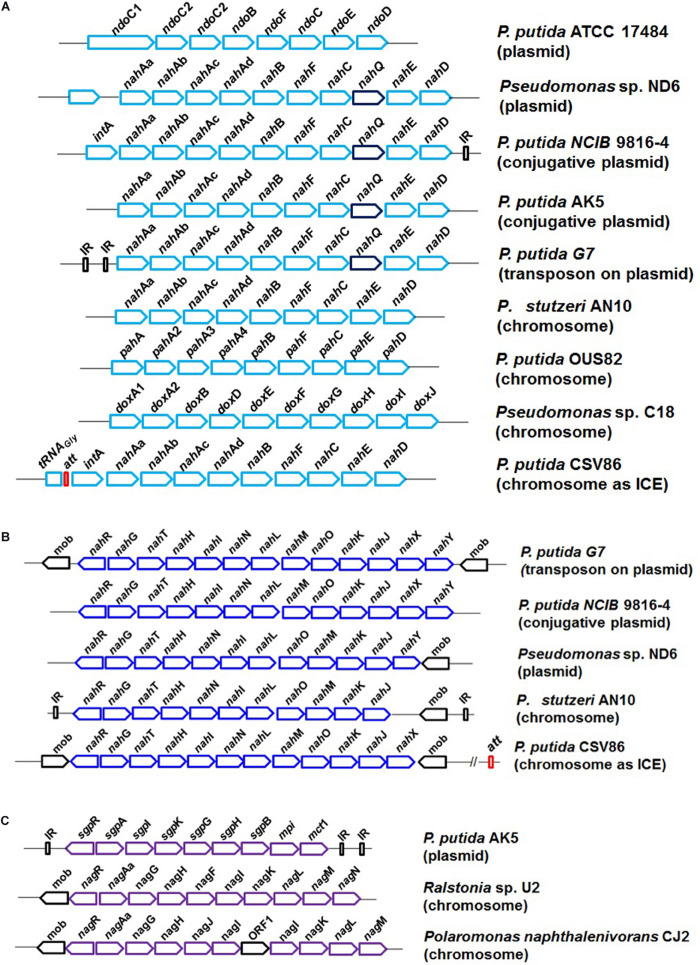
The genetic organization and diversity observed in the degradation of naphthalene in bacterial members; **(A)** naphthalene upper pathway, naphthalene to salicylate metabolism; **(B)** naphthalene lower pathway, salicylate to central carbon pathway *via* catechol; and **(C)** salicylate to central carbon pathway via gentisate.

Biochemical studies, enzyme regulation pattern, and genetic analyses have shown that the naphthalene degradation genes are arranged as two inducible regulatory units, “operons”: *nah* (“upper pathway,” naphthalene to salicylate) and *sal* (“lower pathway,” salicylate to central carbon pathway *via* catechol). Salicylic acid and its analogs act as inducers (Shamsuzzaman and Barnsley, 1974). The operons are suppressed in the presence of glucose or organic acids. The complete genetic organization of naphthalene degradation (as operons) is summarized in Figure 5. Several nomenclatural variations/forms of *nah* genes (*ndo*/*pah*/*dox*) are described and found to share high sequence homology (90%) in all *Pseudomonas* spp. (Abbasian et al., 2016). The naphthalene “upper pathway” genes are often arranged as *en bloc* in consensus order as shown in Figure 5A. An additional gene *nah*Q has also been reported to be involved in naphthalene metabolism and is often found in between *nah*C and *nah*E, but its actual function is yet to be examined. Similarly, the *nah*Y gene responsible for naphthalene-responsive chemotaxis is found to be present at the distal end of the *nah* operon in some of the members. In *Ralstonia* sp. U2, genes encoding glutathione-*S*-transferase (*gsh*) were found to be present in between *nah*Aa and *nah*Ab without affecting naphthalene utilization trait (Zylstra et al., 1997).

The “lower pathway” (*sal* operon) often consists of *nah*GTHINLMOKJ and converts salicylate to pyruvate and acetaldehyde *via* the catechol *meta* ring-cleavage pathway. Gene *nah*G (encoding salicylate hydroxylase) was found to be conserved at the proximal end of the operon (Figure 5B). As compared with other strains of naphthalene degradation, in *P. putida* CSV86, the *nah* and *sal* operons are tandem and in close proximity (∼7.5 kb). In some Gram-negative members like *Ralstonia* sp. U2, *Polaromonas naphthalenivorans* CJ2, and *P*. *putida* AK5, naphthalene is metabolized to central carbon metabolites *via* the gentisate route (as *sgp*/*nag* operon). The genetic cassette is often present as *nag*AaGHAbAcAdBFCQEDJI with *nag*R (encoding LysR type regulator) located at the upstream end (Figure 5C).

**FIGURE 6:**
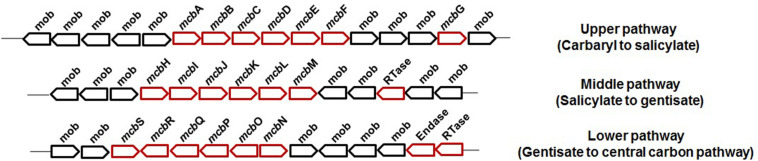
The genetic organization involved in the degradation of Carbaryl by *Pseudomonas* sp. C5pp.

Carbaryl is metabolized to the central carbon cycle *via* 1-naphthol, 1,2-dihydroxynaphthalene, salicylate, and gentisate (Figure 3). Based on genetic and metabolic studies, the pathway has been proposed to be organized into “upper” (carbaryl to salicylate), “middle” (salicylate to gentisate), and “lower” pathways (gentisate to central carbon pathway intermediates) (Singh et al., 2013). Genomic analysis of C5pp (as supercontig-A, 76.3 kb) has shown that genes *mcb*ACBDEF are involved in converting Carbaryl to salicylate, followed by *mcb*IJKL for salicylate to gentisate, and *mcb*OQP for gentisate to central carbon intermediates (as fumarate and pyruvate, Trivedi et al., 2016) (Figure 6).

Enzymes involved in the degradation of aromatics including naphthalene and salicylate are reported to be inducible by the respective compound and suppressed by simple carbon source like glucose or organic acids (Shingler, 2003; Phale et al., 2019, 2020). Among various pathways for the metabolism of naphthalene and its derivatives, regulatory features are studied to some extent for naphthalene and carbaryl. In the case of naphthalene, both “upper” and “lower” pathway genes are regulated through a NahR, which is a LysR-type *trans*-acting positive regulator. It is required for the induction of *nah* genes by salicylate and their subsequent high-level expression (Yen and Gunsalus, 1982). It has been also found that the integration host factor (IHF) along with XylR (sigma-54-dependent transcriptional regulator) are also important for transcription activation of genes in naphthalene metabolism (Ramos et al., 1997). The enzymes of the catechol *meta* ring-cleavage route, i.e., catechol 2,3-dioxygenase and others, were found to be induced in the presence of naphthalene and/or salicylate (Basu et al., 2006). The catechol *ortho* ring-cleavage route enzymes, i.e., catechol 1,2-dioxygenase and others, were found to be induced by benzoate as well as by *cis*,*cis*-muconate (Parsek et al., 1994; Tover et al., 2001).

In strain C5pp, five genes, i.e., *mcb*G, *mcb*H, *mcb*N, *mcb*R, and *mcb*S, code for regulators belonging to the LysR/TetR family transcriptional regulator for Carbaryl degradation. The closest homolog of *mcb*G was found to be LysR-type PhnS (58% amino acid identity) regulator involved in phenanthrene metabolism in *Burkholderia* sp. strain RP00725 (Trivedi et al., 2016). Gene *mcb*H was found to be a part of the middle pathway (salicylate to gentisate) and belongs to the NagR/DntR/NahR-type LysR transcriptional regulators from *Pseudomonas* sp. and *Burkholderia* sp. Members of this family are reported to recognize salicylate as the specific effector molecule to induce the degradation genes. On the other hand, three genes *mcb*N, *mcb*R, and *mcb*S belonging to LysR- and TetR-type transcriptional regulators were identified in the lower pathway (gentisate to central carbon pathway metabolites).

### Genetic Diversity

In prokaryotes, the horizontal gene transfer process (acquisition, exchange, or transmission) through plasmids, transposons, prophages, genomic islands, and integrative conjugative elements (ICEs) is the major reason for the plasticity in bacterial genomes leading to gain or loss of a specific function/property. It provides fast-forward adaptation to various environments, conferring potential adaptive metabolic benefits, viz. aromatic compound degradation, to the bacterial host. The metabolic variations are generally made through the fine-tuning of degradative operons, its regulation, and specificity of the enzymes, aiding in the degradation of a wider range of aromatics (Nojiri et al., 2004; Phale et al., 2019, 2020). The genetic cassette for naphthalene degradation has been found to be located on various mobile elements like plasmids (conjugative and non-conjugative), transposons, genome, ICE, and combinations of different bacterial members (Figure 5). In *P. putida* G7, plasmid NAH7 has *nah* and *sal* operons transcribed in the same direction and is part of a defective transposon that requires Tn4653 transposase for its mobilization (Sota et al., 2006). In *P. putida* strain NCIB9816-4, genes were found to be present on a conjugative plasmid pDTG1 as two operons (∼15 kb apart) which are transcribed in opposite direction (Dennis and Zylstra, 2004). In *P. putida* strain AK5, a non-conjugative plasmid pAK5 codes for enzymes responsible for the degradation of naphthalene *via* the gentisate pathway (Izmalkova et al., 2013). In *P. putida* strain PMD-1, the *nah* operon was found to be located on the chromosome, while the *sal* operon was present on conjugative plasmid pMWD-1 (Zuniga et al., 1981). However, in *P. stutzeri* AN10, all naphthalene degradation genes (*nah* and *sal* operons) were found to be located on the chromosome and hypothesized to be recruited through transposition, recombination, and rearrangement events (Bosch et al., 2000). In *P. putida* CSV86, *nah* and *sal* operons are located on the genome as ICE (ICE*_CSV__86_*). The structure is found to be guarded by tRNA_Gly_ followed by direct repeats denoting sites for recombination/attachment (*att*R *and att*L) at both ends and phage-like integrase, hence structurally similar to the ICE*clc* element (ICE*clc*B13 of *Pseudomonas knackmusii for* chlorocatechol degradation). It has been reported that the genes on the ICE are transferrable through conjugation at a very low (10^–8^) frequency, thus disseminating degradation traits to the recipient (Basu and Phale, 2008; Phale et al., 2019).

Genes responsible for Carbaryl degradation are mostly located on plasmids. *Arthrobacter* sp. RC100 harbors three plasmids (pRC1, pRC2, and pRC300) of which two conjugative plasmids, pRC1 and pRC2, encode enzymes for the conversion of carbaryl to gentisic acid. On the other hand, enzymes involved in the conversion of gentisic acid to central carbon metabolites are located on the chromosome (Hayatsu et al., 1999). A *Rhizobium* sp. strain AC100, which transforms carbaryl to 1-naphthol, harbors a plasmid pAC200 carrying the gene *ceh*A encoding CH as part of the *Tn*ceh transposon flanked by insertion element-like sequence (*ist*A and *ist*B) (Hashimoto et al., 2002). In *Sphingomonas* sp. strain CF06, Carbaryl degradation genes were proposed to present on five plasmids: pCF01, pCF02, pCF03, pCF04, and pCF05 with high inter-DNA homology, indicating gene duplication events (Feng et al., 1997). In one of the Carbaryl-degrading consortia of two *Pseudomonas* spp., the strain 50581 harbors a conjugative plasmid, pCD1 (50 kb), which encodes the *mcd* gene for Carbaryl hydrolase, while the chromosomal counterpart in 50552 encodes enzymes for 1-naphthol degradation (Chapalamadugu and Chaudhry, 1991). The *mcd* gene for carbofuran hydrolase is found to be located on a 100-kb plasmid (pPDL11) in *Achromobacter* sp. strain WM111. This gene was shown to be present on various plasmids (100, 105, 115, or 124 kb) in many bacteria from geographically distant areas (Parekh et al., 1995). In *Pseudomonas* sp. C5pp, all genes responsible for Carbaryl degradation were found to be located on the genome spanning 76.3 kb sequence (Trivedi et al., 2016). The genome (6.15 Mb) analysis revealed the presence of 42 MGEs and 36 GEIs, out of which 17 MGEs were located in supercontig-A (76.3 kb) with mean skewing of G + C content (54–60 mol%), indicating the possible occurrence of HGT events (Trivedi et al., 2016). *P. putida* XWY-1 has shown a similar gene arrangement for Carbaryl degradation, but the genes are located on the plasmid (Zhu et al., 2019).

### Microbial Response(s) for Assisting Efficient Degradation

**FIGURE 7:**
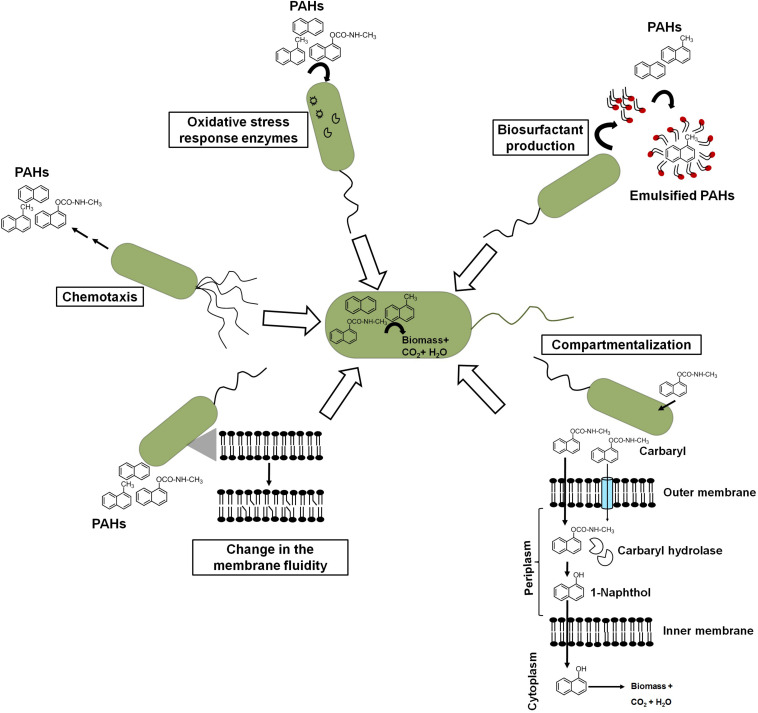
Different cellular response strategies of an ideal aromatic degrading bacterium for efficient biodegradation of xenobiotic pollutants.

Besides metabolic efficiency at the biochemical and genomic levels, microbes do show additional properties or responses like chemotaxis, cell surface alteration properties, compartmentalization, preferential utilization, biosurfactant production, etc. which help them to metabolize these aromatic pollutants from the contaminated niche more efficiently (Figure 7).

#### Chemotaxis

Chemotactic responses have been ascribed to enhance the degradation of organic pollutants in heterogeneous contaminated ecosystems. Pedit et al. (2002) demonstrated that chemotaxis to naphthalene by *P. putida* G7 has increased the rate of naphthalene degradation in an aqueous system. The wild-type strain G7 degraded naphthalene at a much faster rate than chemotaxis-deficient mutant strains. The protein NahY (538 amino acids with a membrane topology) was found to be co-transcribed with *meta*-cleavage pathway genes from the NAH7 plasmid and resembled a chemotaxis transducer protein, which appears to function as a chemoreceptor for naphthalene degradation (Grimm and Harwood, 1997). Another study by Hanzel et al. (2011) showed the chemotactic response of *P. putida* to vapor-phase naphthalene, where gas-phase diffusion resulted in steady naphthalene flux to the cells controlling the chemotactic response of cells. Researchers have taken advantage of such chemotactic behavior to engineer microbes to enhance the degradation rate. It has been shown that chemosensory pathways also regulate other cellular functions like cell division, cell cycle regulation, and biofilm formation, thus contribute in controlling degradation rate. However, the exploration of such trait (chemotaxis) for effective degradation is impeded by certain bottlenecks. The major obstacles are as follows: (a) the same compound/ligand recognition by different paralogous receptors; (b) the presence of other receptors, i.e., energy taxis; (c) the significant sequence divergence in the sensor domain of the same receptor family; and (d) the lack of information on major bacterial sensor proteins (Ortega et al., 2017; Martin-Mora et al., 2018). Sometimes, biodegradation of aromatics results in several metabolites/intermediates that might be chemotactic to one population but repellent to others and, hence, creates further complexity. For the identification of ligand (aromatic)–chemoreceptor interactions, hybrid sensor proteins (PcaY, McfR, and NahY) were constructed by fusing sensor domains and signaling domains of *P. putida* and *Escherichia coli* targeting receptors for aromatic acids, TCA intermediates, and naphthalene (Luu et al., 2019).

#### Cell Surface/Membrane Fluidity

Crucial changes in the structure and integrity of the bacterial membrane were observed in microbes in response to naphthalene and other PAHs. Naphthalene is found to interact through hydrophobic interaction and disturbs the acyl chain interaction, thus increasing the swelling of the membrane and fluidity (Sikkema et al., 1995). To counter the deleterious effect, bacteria regulates membrane fluidity by changing the proportion and composition of fatty acid between *iso/ante-iso* branched chain fatty acids and isomerization of *cis* unsaturated fatty acids to the corresponding *trans* isomers (Heipieper and de Bont, 1994). In *P. stutzeri* when grown under naphthalene amendments, the ratio of saturated to unsaturated fatty acids was found to increase from 1.1 to 2.1, while in *Pseudomonas* sp. JS150, it increased from 7.5 to 12.0 (Mrozik et al., 2004). When grown on naphthalene, *Achromobacter* sp. KAs 3–5 cells showed cellular aggregation around naphthalene crystals with decreased cell surface charge (from −22.5 to −2.5 mV) with cytoplasmic condensation and vacuolation, suggesting changes in the cell structure and cell surface properties (Mohapatra et al., 2019). Although cellular/surface changes are directly linked to better uptake of the aromatic pollutants, no concurrent bioengineering strategies have been thoroughly optimized. The manipulation of cell shapes has been rarely exploited to optimize bioprocesses (Volke and Nikel, 2018). Gene deletions affecting the cell division induce morphological changes in cells. Gene deletions affecting cell division induce morphological changes in cells. In *Bacillus subtilis*, the cell septation protein SepF has shown to be involved in the septum formation and is required for a later step in cell division but does not represent an essential gene. Deletion of genes encoding peptidoglycan hydrolases in *B. subtilis* resulted in elongated cells with increased specific growth rate and improved enzyme production capacities (Cui et al., 2018).

#### Compartmentalization of the Pathway

Compartmentalization of the carbaryl degradation pathway has been proposed for efficient degradation in *Pseudomonas* sp. strains C5pp and C7 (Kamini et al., 2018). Carbaryl is proposed to be transported *via* partitioning in the outer membrane and/or through diffusion porins into the periplasmic space. CH, a periplasmic enzyme, catalyzes the hydrolysis of Carbaryl to 1-naphthol, which is more recalcitrant, hydrophobic, and toxic. Localization of CH in the periplasm with lower affinity for Carbaryl provides controlled formation of 1-naphthol, thus preventing its accumulation and subsequent toxicity to the cells (Kamini et al., 2018). The generated 1-naphthol gets transported *via* partition and/or diffusion across the inner membrane to the cytosol which gets hydroxylated by high-affinity 1NH enzyme to 1,2-dihydroxynaphthalene, which is further metabolized to the central carbon pathway.

#### Preferential Utilization of Aromatic Compounds

Though microbes possess genetic and metabolic abilities to degrade xenobiotics as the carbon source, the hierarchy in utilization, i.e., preferential utilization of simple carbon sources over the complex ones, has been a major barrier for biodegradation. The presence and utilization of simple carbon sources represses genes encoding enzymes for the degradation of complex/non-preferred carbon sources like PAHs. A well-studied example is the utilization of glucose prior to lactose when both were given together to *E. coli* (Jacob and Monod, 1965). *Pseudomonas* are ubiquitously reported to degrade various PAHs and xenobiotic compounds as the carbon source. The carbon source utilization hierarchy in pseudomonads is organic acids > glucose > aromatic compounds (Hylemon and Phibbs, 1972; Collier et al., 1996). However, there is an exception to this. Interestingly, *P. putida* CSV86 displays a unique hierarchy with preferential utilization of aromatics (benzoate, naphthalene, etc.) over glucose and co-metabolism of aromatics with organic acids (Basu et al., 2006). In this bacterium, aromatic degradation and transport genes are not suppressed even in the presence of a second carbon source like glucose or organic acid. The glucose transport and metabolic genes are observed to be repressed when grown on glucose + aromatics, where aromatics get utilized in the first log phase followed by glucose in the second log phase (Basu et al., 2006; Choudhary et al., 2017). On the other hand, the presence of organic acids did not affect the expression of aromatics metabolism, thus making this bacterium a promising candidate for biodegradation studies (Phale et al., 2020).

#### Oxidative Stress Management

It is well established that hydrocarbon biotransformation leads to oxidative stress and upregulation of antioxidant enzymes in microbes. Inefficient naphthalene biodegradation, either by stationary-phase cells or in the presence of toxic compounds, leads to the generation of ROS (Kang et al., 2006). As naphthalene degradative enzymes harbor Fe-S clusters, under oxidative stress, the Fe from haem and Fe-S proteins can be oxidized, which may lead to protein inactivation. Along with superoxide dismutase (SOD), ferredoxin-NADP^+^ reductase (Fpr) mediates reversible redox reactions between NADP^+^/NADPH and two molecules of ferredoxin or flavodoxin, thus scavenging the ROS and repairing Fe-S centers under oxidative stress (Lee et al., 2006). It has been reported that both Fpr and SodA (SOD) are inducible under oxidative stress in *Pseudomonas*, whereas increased activity of SOD and catalase has been observed during growth of four *Pseudomonas* spp. (O1, W1, As1, and G1) in naphthalene-amended condition (Kang et al., 2006). The addition of antioxidants like ascorbate or ferrous iron (Fe^2+^) has been found to confer higher growth rates on naphthalene. In *Rhodococcus erythropolis*, growth on naphthalene showed increased transcription of oxidative stress-related genes of cytochrome P450: *sodA* (Fe/Mn superoxide dismutase), *sod*C (Cu/Zn superoxide dismutase), and *rec*A (Sazykin et al., 2019). Comparative quantitative proteomic analysis of naphthalene-grown cells of *Pseudomonas* sp. showed upregulation of various oxidative stress response-related proteins as a strategy to tackle the stress (Herbst et al., 2013).

#### Biosurfactant Production

In response to hydrophobic carbon sources, microbes have been reported to produce biosurfactants which are amphiphilic, surface-active compounds and assemble at the oil–water or air–water interface. This aids in pseudo-solubilization and facilitates the uptake of aromatics for efficient biodegradation (Rahman et al., 2002). Due to these properties, biosurfactants have a wide application in various industries. The addition of chemical surfactant or biosurfactants to bacterial culture displayed increased efficiency and rate of degradation of hydrocarbons. Among biosurfactants, rhamnolipids produced by *Pseudomonas aeruginosa* are well studied and characterized (Hisatsuka et al., 1971; Rahman et al., 2002). Besides, other types of biosurfactants include lipopeptide (viscosin from *P. fluorescens*), Emulsan 378 (*P. fluorescens*) (Rosenberg and Ron, 1999), trehalose dimycolipids from *Rhodococcus* sp. (Ramdahl, 1985), lichenysins from *Bacillus* sp. (Saraswathy and Hallberg, 2002), and surfactin from *B. subtilis* (Siegmund and Wagner, 1991) and *B. amyloliquefaciens* (Zhi et al., 2017). These potent surfactants are shown to reduce surface tension from 72 to <30 dyn/cm, thus resulting in better uptake of hydrocarbons. The production of various rhamnolipid- and glycolipid-based biosurfactants has been reported for species members of *Pseudomonas*, *Bacillus*, *Rhodococcus*, *Burkholderia*, etc. while growing under naphthalene and methylnaphthalenes (Kanga et al., 1997; Puntus et al., 2005). *P. maltophilia* CSV89 when grown on aromatic compounds like naphthoic acid produced an extracellular biosurfactant, Biosur-Pm (Phale et al., 1995). The kinetics of Biosur-Pm production suggested its synthesis to be a growth- and pH-dependent process. Cells were found to produce a higher amount of Biosur-Pm at neutral pH as compared with pH 8.5. Cells grown at pH 8.5 were more hydrophobic with higher affinity toward aromatic and aliphatic compounds as compared with cells grown at pH 7.0. In *Rhodococcus* sp. N6, a higher carbon to nitrogen ratio (C:N) and iron-limiting conditions are responsible for the optimum production of extracellular biosurfactants (Mutalik et al., 2008). Attempts have been made for the enhanced biosynthesis of biosurfactant (surfactin) through the optimization of bacterial strains and fermentation. However, the low surfactant titers (1.0 g/L) in the medium are a challenge to produce at a large scale (Jiao et al., 2017; Wu et al., 2019). As a result, genetic engineering methods have been used to enhance its biosynthesis. However, due to a large-size operon (∼25 kb) and complex biosynthetic regulation of quorum sensing system, it has shown to be difficult to engineer (Jiao et al., 2017; Wu et al., 2019). *Bacillus* spp. have been engineered to improve surfactin production mainly through promoter exchanges (*srf*A operon), overexpression of the surfactin exporter YerP, and the regulators ComX and PhrC (Jiao et al., 2017). However, these genetic engineering methods all resulted in a single or a few gene modifications, but the commercial production has still not been achieved. Hence, further knowledge-based optimizations need to be explored.

## Factors Affecting the Biodegradation of PAHS

The PAH biodegradation studies have been mainly investigated under standard laboratory conditions. However, at the contaminated site or environment, numerous abiotic and biotic factors (temperature, pH, oxygen, nutrient availability, substrate bioavailability, other xenobiotics, end-product inhibition, etc.) are shown to vary and influence the degradation ability of microorganisms.

Temperature has a profound effect on the biodegradation of PAHs. With increasing temperature, the concentration of dissolved oxygen decreases, which impacts the metabolism of aerobic microbes, as they require molecular O_2_ as one of the substrates for oxygenases which perform ring-hydroxylating or ring-cleaving reaction. It is often noted that the increase in temperature transforms parent PAHs into more toxic compounds, which inhibits biodegradation (Muller et al., 1998).

It has been observed that many of the PAH-contaminated sites have extreme pH conditions, for example, acidic mine drainage-impacted sites (pH 1–4) and alkaline leachate-impacted gas/coal gasification sites (pH 8–12). These conditions are found to impact biodegradation processes severely. Hence, the addition of suitable chemicals (having mild to minimum oxido-reductive potential) is recommended to adjust the pH before application of microbes for bioremediation like ammonium sulfate or ammonium nitrate for alkaline soil and liming with calcium or magnesium carbonate for acidic sites (Bowlen et al., 1995; Gupta and Sar, 2020).

The availability of oxygen at impacted sites is a rate-limiting factor for biodegradation of PAHs. Owing to the oxido-reductive environmental condition, oxygen is often introduced from an external source (tilling, air-sparging, and addition of chemicals) for the *in situ* bioremediation process (Pardieck et al., 1992). Odencrantz et al. (1996) have shown the effective bioremediation of BTEX compounds in a contaminated aquifer after the addition of magnesium peroxide (O_2_-releasing compound). Another study involving *in situ* degradation of phenols and BTEX in a contaminated aquifer has used sodium nitrate through injection and construction of abstraction wells for effective bioremediation (Bewley and Webb, 2001).

The availability of nutrients like N, P, K, and Fe is reported to be essential for effective bioremediation. Thus, supplementation of these nutrients (referred to as biostimulation) in limiting concentration is required to enhance the growth of indigenous microorganisms for effective bioremediation of PAHs (Sarkar et al., 2020). On the other hand, high/excess nutrient levels were found to affect the rate of biodegradation of PAHs.

The bioavailability of PAHs in the environment is often limited due to their low aqueous solubility (hydrophobicity) and strong tendency to adsorb onto minerals and organic matter of the matrix (Cornelissen et al., 2005; Rein et al., 2016). The aqueous solubility of PAHs decreases with increasing molecular weight, which in turn reduces the bioavailability in groundwater and surface water. It has also been shown that aging (retention for a longer time in the environment) of PAHs renders them more difficult to extract/degrade from a polluted ecosystem, thus significantly impacting the rate of bioremediation (Luo et al., 2012).

Contaminated environments are usually burdened with dynamic concentrations of a particular PAH or combination/mixtures of PAHs, which often limits the degradation efficiency (Penning et al., 1999; Juhasz and Naidu, 2000). The presence of a high concentration of naphthalene has shown inhibitory effects on the degradation of other PAHs by bacterial co-cultures (Bouchez et al., 1995). Phenanthrene biodegradation is reported to be inhibited due to the presence of a higher concentration of naphthalene and methylnaphthalene (Stringfellow and Aitken, 1995). In some environments, the physicochemical transformation processes (photo-oxidation, chemical oxido-reduction) generate more toxic by-products, which limit the metabolism of PAHs. Hence, both the amount and composition (concentration) of PAHs as well as the toxic (by-)products generated during treatment at the contaminated sites should be monitored before strategizing the effective *in situ* bioremediation plans.

Although much information on pollutant-degrading bacterial strains, metabolic steps, and enzymes is involved, the field-scale application and bioremediation attempts have yielded poor results. Such failures can be attributed due to lack of insights into important factors such as (i) the thermodynamic feasibility of assembled catabolic networks, (ii) kinetic properties and regulatory feature of the enzymes, (iii) physicochemical properties of metabolites, (iv) extent of induction of enzymes, (v) suppression of degradation pathways by a simple carbon source like carbohydrates and organic acids, (vi) cross-talk between metabolic routes, and (vii) stress responses and changes in overall cell physiology (de Lorenzo, 2009; Ramos et al., 2011; Phale et al., 2020). In this context, the selection of suitable candidate(s) for biodegradation is a crucial initial step. Over the years, several microbes have been considered for application in biodegradation processes, but no single naturally isolated bacterial strain was found to possess all the desired characteristics. Hence, metabolic engineering strategies for optimizing genetic and regulatory processes are becoming suitable alternatives to improve overall cell robustness and biodegradation rate (Nielsen et al., 2014). Recent advancement in the use of systems biology and “omics” techniques has helped to gain a deeper insight into the genetic and physiological background of microbes to model enzymatic reactions and determine the constraints for efficient elimination. To overcome environmental constraints (factors), computational tools/databases are assisting in the fine-tuning of pathways to maximize performance in a cost-effective manner (Dvorak et al., 2017). In addition, researchers are developing effective consortia which are shown to degrade various pollutants based on co-metabolism/synergistic actions of different mutualistic/symbiotic microbes. Further research on genome-scale metabolic modeling (fluxomic and interactomic) studies enabled us to unravel the biodegradative behavior of isolates. In the last decade, BESs including microbial fuel cells and microbial electrolysis cells have become preferred options for waste treatment at the industrial scale. By integrating biological treatment, electrolytic dissociation, and electrochemical oxidation/reduction, these systems are regarded as a new sustainable strategy for the treatment of various organic pollutants with higher treatment efficiencies than conventional processes (Huang et al., 2011; Pant et al., 2012; Nazari et al., 2020). Especially, the oxidation of organic pollutants in wastewater of domestic, brewery, and paper-recycling units, food processing industry, and landfill leachate has been successful (Nazari et al., 2020). Besides, the catalytic activities of electrochemically active microorganisms lead to sustainable bioprocessing approaches, viz. electricity generation, nutrient recovery, formation of value-added by-products, and bioremediation (dehalogenation and reduction of nitro-organics). With the knowledge of the participating microbes in biodegradation and their metabolic potential, several microbial bioremediation approaches such as biostimulation and bioaugmentation are popularized, which are elaborated further in the next section. Along with microbes, the introduction of plants to the polluted site(s) and the use of plants with microbes as well as composting and biopiling are increasingly being preferred for contaminant attenuation in polluted sites.

## Bioremediation Strategies

**FIGURE 8:**
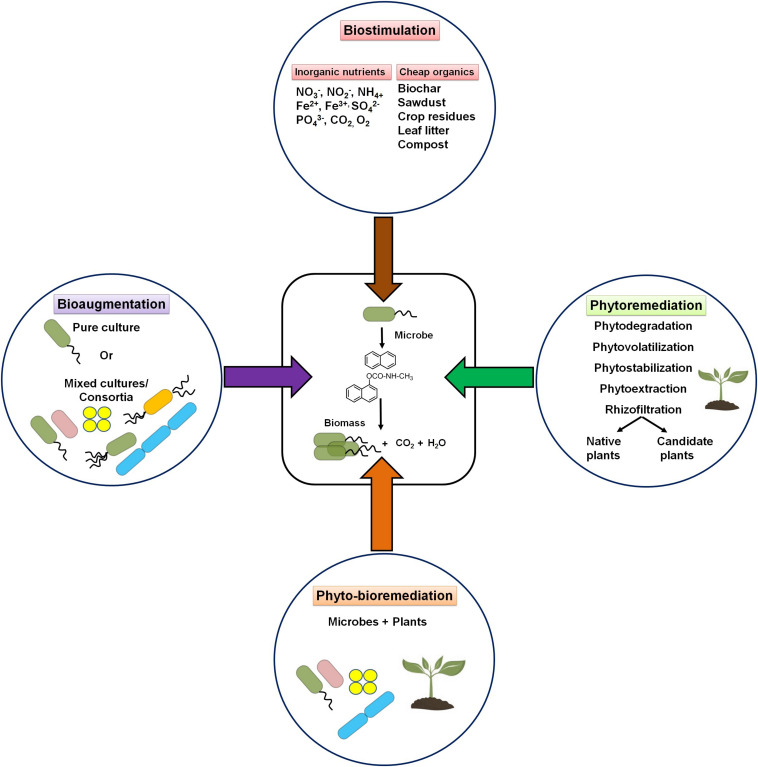
Schematic diagram showing various bioremediation strategies for degradation/cleanup of xenobiotic aromatic pollutants.

A contaminated environment is usually found to be deficient in essential nutrients to support microbial growth and metabolic processes of potential microbes. Based on the knowledge of participating microbial members (or communities) and their nutrient requirement, two major strategies are popularly used for the bioremediation of contaminated niches, i.e., biostimulation and bioaugmentation. In addition, the use of plants (phytoremediation) either alone or in combination with potential microbe(s) (phyto-bioremediation) is also preferred as an effective cleanup procedure. The strategies are depicted in Figure 8.

Biostimulation refers to the supplementation of one or more nutrients to the impacted sites for accelerating the growth and metabolic abilities of microorganisms (Sarkar et al., 2020). The application of water-soluble inorganic nutrients (NO_2_^–^, NO_3_^–^, Fe^3+^, SO_4_^2–^, and CO_2_), slow-release fertilizers, and oxygenation resulted in the increase of the rate of degradation (Sarkar et al., 2016, 2020). It has been estimated that an optimal ratio of 100:10:1 for C:N:P is required for effective biodegradation of hydrocarbon pollutants (Dibble and Bartha, 1979; Dias et al., 2012). Several laboratory studies have shown that excess carbon (as in PAHs) compared with other nutrients critically affects the microbial metabolism of hydrocarbons (Sarkar et al., 2020). Several low-cost nutrients like biochar, sawdust, crop residues, leaf litter, compost, etc. have been successfully applied to enhance the growth of microorganisms and remediate contaminated sites (Simons et al., 2013; Qin et al., 2013). Among all nutrients, nitrate amendment has been the most effective biostimulating agent for bioremediation of hydrocarbon pollutants (Roy et al., 2018). Effective biodegradation (80%) of petroleum sludge containing 400 g/kg total petroleum hydrocarbons (TPH) was achieved with nitrate amendment. Increased activity (stimulation) of nitrate-reducing *Azovibrio*, *Pseudoxanthomonas*, *Commamonas*, and *Bacillus* was evident in this study (Sarkar et al., 2016; Roy et al., 2018). Other studies have also shown a positive effect of either N or P on the biodegradation of pesticides (Sharma et al., 2019).

Bioaugmentation aims to supplement microbes externally, either as a single pure culture or mixed culture (consortium), to enhance the overall metabolic activity for complete degradation. Pure cultures of *Pseudomonas*, *Flavobacterium*, *Sphingomonas*, *Achromobacter*, *Bacillus*, and *Rhodococcus* are promising bioaugmentation agents. It is observed that the use of mixed bacterial culture is more advantageous than pure culture due to synergistic interactions among microbial species (Tyagi et al., 2011; Ghaly et al., 2013; Sarkar et al., 2020). The use of consortium harboring *Delftia*, *Bacillus cereus*, *Pseudomonas resinovorans*, *P. fluorescens*, *Exiguobacterium*, *Arthrobacter*, and *R. erythropolis* has been effective for the bioremediation of diesel-contaminated soil (Sprocati et al., 2012). A consortium of *Aeromonas*, *Alcaligenes xylosoxidans*, *Gordonia*, *P. fluorescens*, *P. putida*, *Rhodococcus equi*, *S. maltophilia*, and *Xanthomonas* has shown 89% degradation of various hydrocarbons in a soil column within a year (Szulc et al., 2014). A combinatorial approach where both biostimulation and bioaugmentation are applied led to a more effective bioremediation of PAHs and short- and long-chain alkanes for various hydrocarbon-impacted niches (Sarkar et al., 2016; Wu et al., 2016). The use of microbial consortium harboring various *Pseudomonas* spp. and other *gamma-proteobacteria* members in combination with NH_4_NO_3_ and K_2_HPO_4_ is an effective strategy for total petroleum hydrocarbon degradation (Varjani et al., 2015).

Plants (native and adapted species) are also used to remove aromatic pollutants from contaminated sites and are referred to as phytoremediation. Moreover, it particularly suits to the treatment of larger areas of surface contamination, where other methods may not be as effective. Several species of either native plants or grasses (*Agropyron*, *Bouteloua*, *Cyanodondactylon*, *Elymus*, *Festuca*, *Melilotus*, etc.) or legumes (*Alfalfa*) are popularly used to degrade various PAHs (Harvey et al., 2002; Hall et al., 2011; Truu et al., 2015). A combined use of different types of grasses was shown to be effective for phytoremediation of various PAHs and pesticides in sandy loam soils (Lalande et al., 2003; Basumatary et al., 2012). These plants have also shown to release various metabolites (amino acids, sugars, inorganic nutrients) and enzymes (dehalogenase, reductase, peroxidase, laccase, etc.) in the root exudates. This helps to enhance the growth and metabolic activities of rhizospheric microbes, or they interact directly with PAHs to biotransform. Various studies have also shown that the combined use of plants and microbes is an emerging cost-effective technique for contaminant removal for maximum efficiency with minimum environmental disturbances (Figure 8). The application of such techniques with agricultural management methods like integrated nutrient management, water use, crop rotation, and other agronomic practices will help in the complete removal/cleaning up of such pesticide pollutants as well as xenobiotics from the agricultural fields and contaminated site.

## Conclusion and Future Direction

The contaminated ecosystems harbor vast microbial diversity with dynamic metabolic flexibility. However, it often lacks suitable conditions, i.e., robust microbial members with metabolic pathways and its regulation for complete mineralization, nutrients required for metabolism, and other biotic/abiotic factors. Hence, designing appropriate microbial host(s) with efficient, rapid, and broad range of degradation abilities is a key factor in the scaling up of the bioremediation process. In addition, other properties like chemotaxis, cell surface/membrane fluidity, compartmentalization of pathway, and biosurfactant production are advantageous for the enhanced metabolism of PAHs. Furthermore, organisms capable of degrading a broad range of PAHs with genotypic and phenotypic stability of degradation trait, i.e., chromosomal origin and microbes with preferential utilization of such pollutants even in the presence of a simple carbon source like carbohydrates or organic acids, are of immense importance for efficient bioremediation. The use of next-generation and high-throughput molecular and computational tools is most valuable for understanding system biology of suitable xenobiotic degrading microbes. Since natural bioremediation is a slow and complex process, a system biology-based platform will be of much value for metabolic engineering (patchwork assembly, gene shuffling, and genome editing) of microbes for the reconstruction of new pathways and robust microbial chassis. The use of BESs incorporating metabolically robust microbial hosts as microbial fuel cells and microbial electrocatalytic cells might be an alternative approach for the treatment of a wider varieties of pollutants. The combined implication of omics—genomics, metagenomics, metagenome-assisted genome assemblies, transcriptomics, proteomics, metabolomics, and phenomics—would further aid in understanding the complex behavior of microbes, fine-tuning of selected biochemical routes, its networks, and whole-cell biocatalysis. In addition, more advanced computational tools are required to fully exploit the omics-derived data for better functional assignments and understanding metabolic fluxes and their interactions. In recent days, adaptive laboratory evolution has been frequently used to transform the desired properties in environmental bacteria and for the fine-tuning of recombinant microorganisms. Exposing microorganisms with desirable target chemical pollutant(s) for prolonged duration enabled the isolation of potent degrading bacteria. Screening and identification of potent plant growth-promoting and pollutant-degrading microbes would be of added value for increasing crop yield/productivity in agroecosystems and the biodegradation of xenobiotics like pesticides simultaneously. The development of integrated platforms which might provide all the information related to bioremediation research, including data, analytical methods, and pipelines, is the current need of the hour. In the context of the use of genetically engineered microbes for pollutant biodegradation, public environmental concerns and regulatory constraints have delimited the field tests of microbes and also have affected the quality and progress of biotechnological research. So, better knowledge dissemination, popularization of achievements, and mass awareness on the beneficial use of engineered microbes must be a priority. Together with this, cross-border coordination through scientists/working groups around the world in different laboratories for data sharing and database maintenance would further help in rationalizing bioremediation plans. The use of cybernetics, artificial intelligence, nano-bioremediation (nanoscale zero-valent metals/nano-organics), agricultural/agronomic management practices, etc. will enable further value addition and cost reduction of sustainable on-site bioremediation strategies in the near future.

## Author Contributions

BM and PP conceptualized, organized, wrote, and edited the manuscript. Both authors contributed to the article and approved the submitted version.

## Conflict of Interest

The authors declare that the research was conducted in the absence of any commercial or financial relationships that could be construed as a potential conflict of interest.

## Footnotes

1 http://www.inchem.org/documents/iarc/vol82/82-06.html

2 https://ntp.niehs.nih.gov/

3 http://ppqs.gov.in/statistical-database
